# Effects of 17β-Estradiol Treatment on Metabolic Function and Aortic Relaxation in Castrated Male Rats

**DOI:** 10.3390/ijms26188885

**Published:** 2025-09-12

**Authors:** Rifat Ara Islam, Md Rahatullah Razan, Ankita Poojari, Mohammad Moshiur Rahman, Hao Wei, Hana S. Alhamadsheh, Melanie Felmlee, Atefeh Rabiee, Mitra Esfandiarei, Roshanak Rahimian

**Affiliations:** 1 Department of Pharmaceutical Sciences, Thomas J. Long School of Pharmacy, University of the Pacific, Stockton, CA 95211, USArazan@wustl.edu (M.R.R.); a_poojari@u.pacific.edu (A.P.); m_rahman4@u.pacific.edu (M.M.R.); h_wei1@u.pacific.edu (H.W.); hanahamadsheh@gmail.com (H.S.A.); mafelmlee@manchester.edu (M.F.); arabiee@pacific.edu (A.R.); 2 Biomedical Sciences Program, College of Graduate Studies, Midwestern University, Glendale, AZ 85308, USA; mesfan@midwestern.edu

**Keywords:** Cardiovascular disease, male-to-female, estrogen-treatment, aortic relaxation, nitric oxide (NO)

## Abstract

Exogenous estrogen use in male-to-female individuals has been linked to increased cardiovascular disease risk, though the mechanisms remain unclear. This study examines the effects of 17β-estradiol (E_2_) on metabolic and aortic function in castrated (CAS) male Sprague Dawley rats. CAS rats received subcutaneous E_2_ (CAS + E_2_) or placebo (CAS + PL) pellets for ~35 days, with intact males serving as controls. Endothelium-dependent vasorelaxation (EDV) in response to acetylcholine and contractile responses to phenylephrine were measured in aorta before and after pharmacological inhibitors. Metabolic parameters and expression of proteins associated with vascular and insulin signaling were also determined in aorta and white adipose tissue (WAT). E_2_ treatment reduced body weight, improved HbA1c and enhanced glucose tolerance in CAS rats compared to the CAS + PL group. Improved glucose homeostasis was associated with upregulation of estrogen receptor alpha, phosphorylated Akt/Akt, and glucose transporter-4 expression in WAT. However, E_2_ increased plasma triglyceride and impaired EDV, indicating compromised vascular function. Our results suggest that impaired aortic relaxation in the CAS + E_2_ group may be partly attributable to increased contractility. Additionally, we observed reduced G protein-coupled estrogen receptor and elevated inducible nitric oxide synthase expression, warranting further investigation into whether these factors contribute to the effects of E_2_ on aortic relaxation.

## 1. Introduction

Cross-sex hormone therapy (CSHT) plays an essential role in managing gender dysphoria among transgender population. However, our understanding of the impact of CSHT on cardiovascular health of the transgender population remains limited [1,2]. Recent studies indicate that the use of estrogen in male to female (MtF) individuals is associated with an elevated risk of myocardial infarction and ischemic stroke [3]. Furthermore, it has been shown that CSHT in MtF population can lead to an increase in body mass index (BMI), worsen insulin resistance and elevate the risk of diabetes [4,5,6,7]. Moreover, a meta-analysis reported that CSHT resulted in an increase in triglyceride levels, but did not significantly affect total cholesterol, HDL or LDL levels in MtF population [8]. The impact of CSHT on lipid profile, however, can vary depending on the type of antiandrogen used. For instance, MtF individuals receiving leuprolide had significantly higher total cholesterol and HDL levels, while those on cyproterone acetate showed opposite effects. Both of the antiandrogens were used in combination with transdermal estradiol [9]. Therefore, to eliminate the confounding influence of antiandrogens and solely focus on the effects of estrogen, we conducted our study on estrogen-treated castrated (CAS) male rat model.

A CAS rat model treated with estrogen may serve as a valuable model of MtF transition for elucidating the mechanisms underlying altered metabolic function. In the current study, we hypothesized that treatment with 17β-estradiol (E_2_) exacerbates the risk of cardiovascular disease (CVD) in CAS male rats by promoting metabolic syndrome and impairing vascular function. Specifically, the first aim of this study was to investigate the impact of E_2_ treatment on key metabolic parameters in plasma and the endothelium-dependent vasorelaxation (EDV) in aortic tissues taken from CAS male rats. EDV is a reliable and reproducible parameter to assess endothelial function under pathological conditions [10]. In the current study, we assessed EDV by measuring the acetylcholine (ACh)-induced aortic relaxation in phenylephrine (PE)-precontracted aortic rings taken from E_2_-treated CAS male rats.

White adipose tissue (WAT) is one of the key organs involved in insulin-mediated glucose uptake [11,12,13]. Estrogen treatment has been shown to enhance glycemic control by activating estrogen receptor alpha (ERα) in WAT [14]. In contrast, estrogen receptor beta (ERβ) appears to exert opposing effects, and it may contribute to a diabetogenic phenotype [15,16]. Hence, our second objective was to investigate the expression of signaling proteins associated with glucose homeostasis in WAT of CAS male rats treated with E_2_.

In addition to ERα and ERβ, G protein-coupled estrogen receptor (GPER or GPR30) has been detected on the aortic endothelial and smooth muscle cells [17]. Previous studies have shown that alterations in ER subtypes along with changes in receptor sensitivity or signaling may contribute to increased oxidative stress and inflammation in the vasculatures of MtF population [18,19,20]. Therefore, we also aimed to measure the expression of these three receptors and assess whether the expression of these proteins has been impacted in the aortic tissues taken from CAS + E_2_ rats. In large artery such as aorta, nitric oxide (NO) is a major mediator of vasorelaxation [21,22,23]. An altered level of NO has been associated with changes in the expression of endothelial nitric oxide synthase (eNOS) [24]. While activation of arterial eNOS is known to provide anti-inflammatory, antithrombotic, and anti-atherosclerotic effects [24,25], the upregulation of inducible nitric oxide synthase (iNOS) has been reported in vascular inflammation [26,27,28]. Thus, the last aim of this study was to assess the effect of E_2_ treatment on the relative contribution of NO to aortic relaxation, as well as the expression of eNOS and iNOS in the aorta of CAS male rats. This study demonstrates that E_2_ treatment in castrated male rats improves metabolic parameters but negatively impacts vascular function.

## 2. Results

### 2.1. Castration and E_2_ Treatment Altered Plasma Testosterone and Estrogen Levels

Plasma testosterone level was undetectable in both CAS + PL and CAS + E_2_ rats as compared to intact male rats (Table 1). Plasma estrogen concentration did not differ between intact male and CAS + PL rats. However, E_2_ treatment increased the level of plasma estrogen in CAS rats. The estrogen level in CAS + E_2_ rats was significantly higher compared to the other two groups of animals (Table 1).

### 2.2. E_2_ Treatment Altered Metabolic Parameters and Glucose Tolerance

Body weight was significantly lower in CAS + E_2_ rats compared to both intact male and CAS + PL groups. The visceral WAT (vWAT) weight was also significantly reduced in CAS + E_2_ rats compared to the other two groups. Nonetheless, the vWAT weight to body weight ratio (%) did not differ among the groups. Similarly, no significant differences were observed in non-fasting blood glucose or plasma insulin levels across the groups. HbA1c level was significantly lower in CAS + E_2_ rats compared to both intact male and CAS + PL rats. However, no differences in body weight, vWAT weight or HbA1c level were observed between intact male and CAS + PL groups, indicating no effect of castration on these parameters (Table 2).

Although plasma HDL level was significantly increased in CAS + E_2_ compared to the intact male and CAS + PL groups, no significant difference in plasma LDL level was observed across the experimental groups. Nonetheless, triglyceride level was significantly higher in CAS + E_2_ rats when compared to the other two groups (Table 2). Notably, while both body weight and vWAT weight were significantly reduced in CAS + E_2_ rats, the vWAT weight-to-body weight ratio (%) remained unchanged, suggesting that the reduction in vWAT weight was proportional to the overall body weight loss rather than representing a selective effect of E_2_ on visceral adiposity.

**Figure 1:**
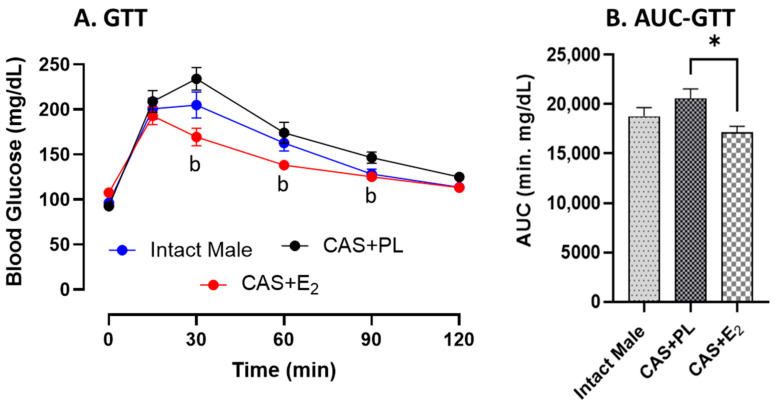
Glucose tolerance is improved in CAS + E_2_ rats. (**A**) Glucose tolerance test (GTT) and (**B**) area under the curve for GTT (AUC-GTT) in intact male (n = 10), castrated placebo (CAS + PL, n = 11) and castrated E_2_-treated (CAS + E_2_, n = 11) rats. Data is expressed as mean ± SEM. ^b^ *p* < 0.05 vs. CAS + PL, analyzed using two-way ANOVA with repeated measure followed by Tukey’s post hoc test. Capped line indicates significant differences between groups. * *p* < 0.05, one-way ANOVA followed by Tukey’s post hoc test.

No difference in glucose tolerance was observed between intact male and CAS + PL rats, as reflected by similar glucose levels (Figure 1A) and area under the curve (AUC) for glucose tolerance test (GTT) (Figure 1B). However, CAS + E_2_ animals exhibited improved glucose tolerance at 30, 60 and 90 min of glucose injection compared to CAS + PL group (Figure 1A), and their AUC-GTT was significantly lower (Figure 1B).

### 2.3. Effects of E_2_ Treatment on White Adipose Tissue Signaling Pathways Involved in Glucose Regulation

**Figure 2:**
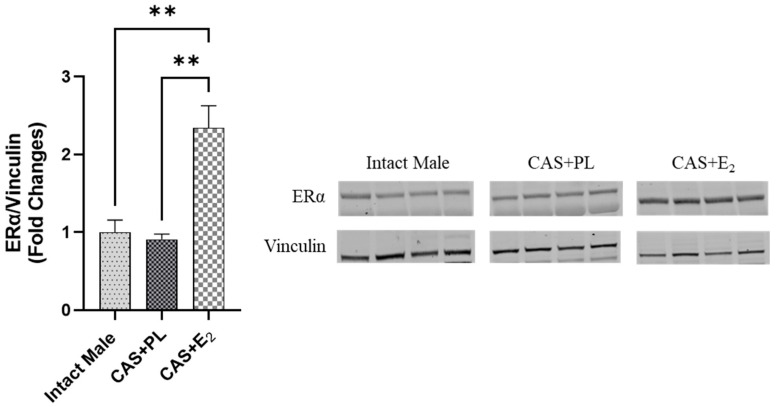
ERα expression is elevated in subcutaneous white adipose tissue (ScWAT) of CAS + E_2_ rats. Western blot analysis of ERα in ScWAT of intact male, castrated placebo (CAS + PL) and castrated E_2_-treated (CAS + E_2_) rats. Protein levels were quantified by densitometric analysis and normalized to corresponding housekeeping protein. Data is expressed as mean ± SEM. n = 4 per group. Each lane represents a sample from a different rat. Bands of target and housekeeping proteins were shown from the same membrane. Capped lines indicate significant differences between two groups. ** *p* < 0.01, analyzed using one-way ANOVA followed by Tukey’s post hoc test. ERα, estrogen receptor α.

**Figure 3:**
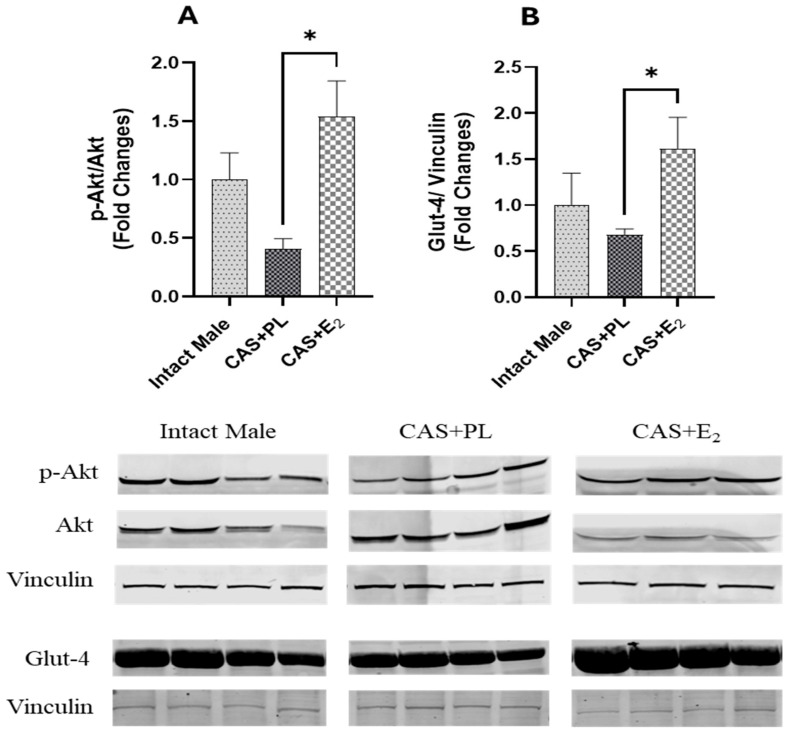
pAkt/Akt ratio and Glut-4 expressions are elevated in subcutaneous white adipose tissue (ScWAT) of CAS + E_2_ rats. Western blot analysis of (**A**) p-Akt/Akt and (**B**) Glut-4 in ScWAT of intact male (n = 4), castrated placebo (CAS + PL, n = 4) and castrated E_2_-treated (CAS + E_2_, n = 3 for p-Akt/Akt and n = 4 for Glut-4) rats. Protein levels were quantified by densitometric analysis and normalized to corresponding housekeeping protein. Data is expressed as mean ± SEM. Each lane represents a sample from a different rat. Bands of target and housekeeping proteins were shown from the same membrane. Capped lines indicate significant differences between two groups. * *p* < 0.05, analyzed using one-way ANOVA followed by Tukey’s post hoc test. p-Akt, phosphorylated Akt; Glut-4, glucose transporter-4.

ERα expression in subcutaneous WAT (ScWAT) was significantly higher in CAS + E_2_ rats compared to both intact male and CAS + PL groups, whereas no difference was observed in ScWAT ERα expression between intact male and CAS + PL groups (Figure 2). However, assessment of ERβ expression in ScWAT was inconclusive (Supplementary Figure S1). The phosphorylated Akt/total Akt (p-Akt/Akt) ratio in ScWAT was significantly elevated in CAS + E_2_ rats compared to the CAS + PL group (Figure 3A). Moreover, E_2_ treatment significantly increased glucose transporter-4 (Glut-4) expression in ScWAT of CAS rats compared to CAS + PL group (Figure 3B).

### 2.4. Effects of E_2_ Treatment on Acetylcholine (ACh)-Induced Aortic Relaxation

**Figure 4:**
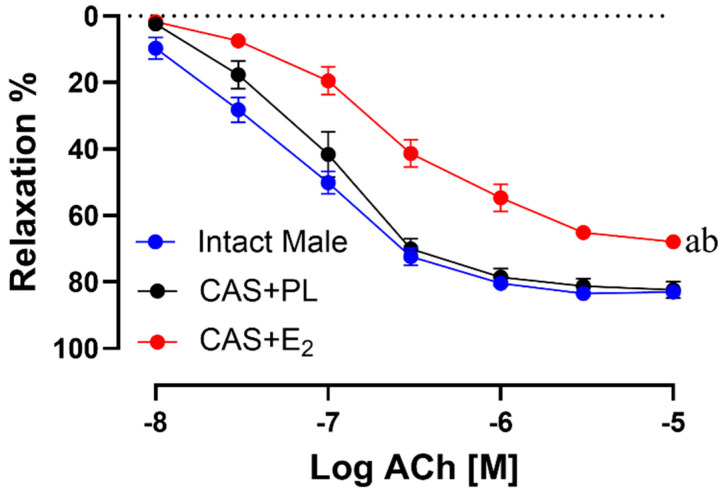
Acetylcholine (ACh)-induced vasorelaxation is reduced in CAS + E_2_ rats. Vasorelaxation responses to cumulative concentrations of ACh (10^−8^ to 10^−5^ M) in intact aortic rings precontracted with phenylephrine (PE, 2 µM) of intact male, castrated placebo (CAS + PL) and castrated E_2_-treated (CAS + E_2_) rats. Data is expressed as mean ± SEM. n = 8 per group, ^a^ *p* < 0.05 vs. intact male, ^b^ *p* < 0.05 vs. CAS + PL, analyzed using two-way ANOVA with repeated measure followed by Tukey’s post hoc test.

ACh-induced vasorelaxation was significantly reduced in CAS + E_2_ rats compared to both intact male and CAS + PL groups (Figure 4). The maximum relaxation response (R_max_) was significantly lower in CAS + E_2_ rats compared to the other two groups (Table 3).

Similarly, sensitivity to ACh (pD_2_: −logEC_50_) was significantly reduced in CAS + E_2_ rats compared to both intact male and CAS + PL groups (Table 3). No significant differences were observed in the concentration response curve (CRC), R_max_ or pD_2_ to ACh in between intact male and CAS + PL groups, indicating castration did not have any impact on the aortic relaxation.

### 2.5. Effects of E_2_ Treatment on Relative Contribution of Cyclooxygenase (COX) Metabolites and Nitric Oxide (NO) to ACh-Induced Aortic Relaxation

**Figure 5:**
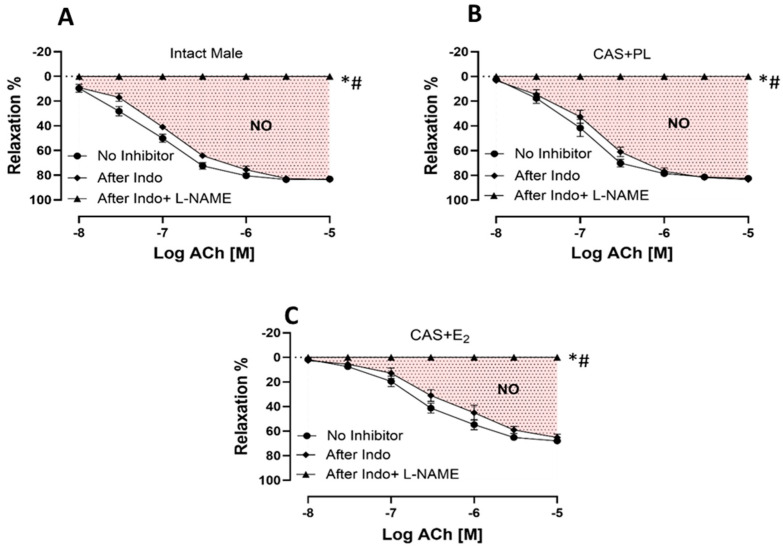
NO-mediated aortic relaxation is reduced in CAS + E_2_ rats. Contribution of cyclooxygenase (COX) metabolites and nitric oxide (NO) to acetylcholine (ACh)-induced relaxation in intact aortic rings precontracted with phenylephrine (PE, 2 µM) of (**A**) intact male, (**B**) castrated placebo (CAS + PL) and (**C**) castrated E_2_-treated (CAS + E_2_) rats. Concentration response curve (CRC) to ACh (10^−8^ to 10^−5^ M) was generated in the absence of inhibitor and then after Indomethacin (Indo, 10 µM), followed by L-NAME (200 µM) in the presence of Indo (10 µM). Data is expressed as mean ± SEM. n = 8 per group, * *p* < 0.05 vs. no inhibitor, # *p* < 0.05 vs. after Indo, analyzed using two-way ANOVA with repeated measure followed by Tukey’s post hoc test. The shaded area represents the contribution of nitric oxide (NO) to vasorelaxation. Indo, Indomethacin; L-NAME, Nω-nitro-L-arginine methyl ester.

To measure the relative contribution of COX-derived metabolites and NO to ACh-induced aortic relaxation in experimental groups, aortic rings were incubated sequentially with Indomethacin (Indo, a COX inhibitor), and Nω-nitro-L-arginine methyl ester (L-NAME, a non-selective NOS inhibitor) in the presence of Indo, using established protocols previously published by our groups and others [29,30,31]. Addition of Indo caused a slight rightward shift of the ACh CRC in all experimental groups, indicating the contribution of relaxing prostanoid prostacyclin (PGI_2_). However, no differences were observed in the CRC to ACh (Figure 5A–C), or in R_max_ and pD_2_ values (Table 4) before and after blocking COX pathway across the three groups of rats. Accordingly, there was no difference in the relative contribution of COX metabolites to vasorelaxation among intact male, CAS + PL or CAS + E_2_ rats, as indicated by the lack of difference in ΔAUC between ACh CRC before and after Indo treatment (Table 4).

The addition of L-NAME in the presence of Indo significantly reduced and ultimately abolished ACh-induced aortic relaxation in all the three experimental groups (Figure 5A–C). The relative contribution of NO to vasorelaxation, reflected by ΔAUC between ACh CRC before and after L-NAME treatment, was significantly lower in CAS + E_2_ rats compared to the other two groups (Table 4). This reduced NO contribution may partly account for the attenuated ACh-induced aortic relaxation observed in CAS + E_2_ rats.

### 2.6. Effects of E_2_ Treatment on Sodium Nitroprusside (SNP)-Induced Aortic Relaxation

**Figure 6:**
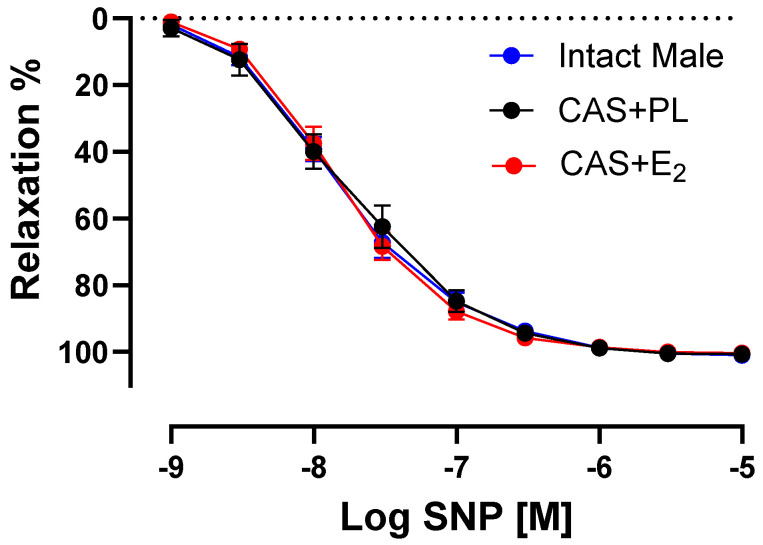
Sodium nitroprusside (SNP)-induced aortic relaxation is similar among groups. Relaxation responses to cumulative concentrations of SNP (10^−9^ to 10^−5^ M) in endothelium denuded aortic rings precontracted with phenylephrine (PE, 2 µM) of intact male, castrated placebo (CAS + PL) and castrated E_2_-treated (CAS + E_2_) rats. Data is expressed as mean ± SEM. n = 8 per group, analyzed using two-way ANOVA with repeated measure.

To determine whether the decreased ACh-induced aortic relaxation in E_2_-treated CAS rats was due to decreased sensitivity of vascular smooth muscle cells (VSMC) to NO, CRC to SNP (a NO donor) was generated in endothelium-denuded rings. No difference was observed in the CRC to SNP, R_max_ or pD_2_ among the three groups of animals, indicating preserved VSMC responsiveness to NO in CAS + E_2_ rats (Figure 6, Table 5).

### 2.7. Effects of E_2_ Treatment on Phenylephrine (PE)-Induced Aortic Contraction

**Figure 7:**
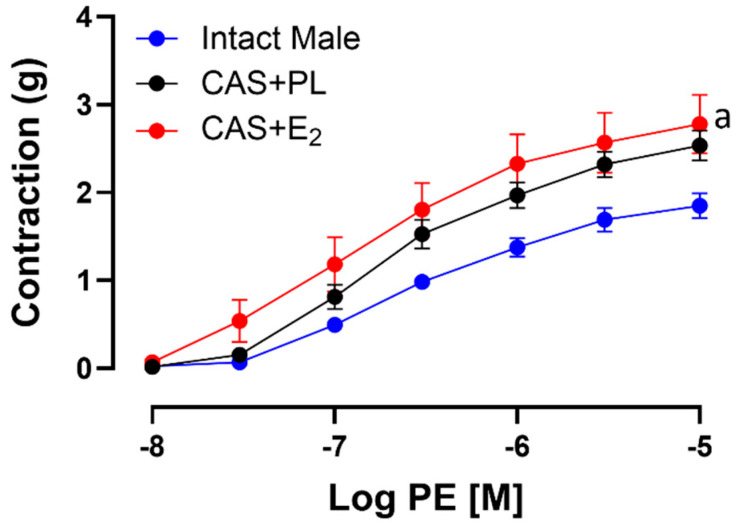
Phenylephrine (PE)-induced aortic contraction is increased in CAS + E_2_ rats. Contractile responses to cumulative concentrations of PE (10^−8^ to 10^−5^ M) in intact aortic rings of intact male, castrated placebo (CAS + PL) and castrated E_2_-treated (CAS + E_2_) rats. Data is expressed as mean ± SEM. n = 8 per group, ^a^ *p* < 0.05 vs. intact male, analyzed using two-way ANOVA with repeated measure followed by Tukey’s post hoc test.

To assess whether the reduced ACh-induced aortic relaxation in E_2_-treated CAS rats was due to increased contractile responses, CRC to PE was generated in experimental groups. Our data indicated that there was no significant difference in CRC, maximum tension (T_max_) or sensitivity (pD_2_) to PE between intact male and CAS + PL rats (Table 6). However, aortic contraction to PE was increased in CAS + E_2_ rats compared to intact males (Figure 7). Our data also showed that sensitivity to PE was unchanged in CAS + E_2_ rats, but this group exhibited a significantly higher T_max_ compared to the intact male rats (Table 6).

**Figure 8:**
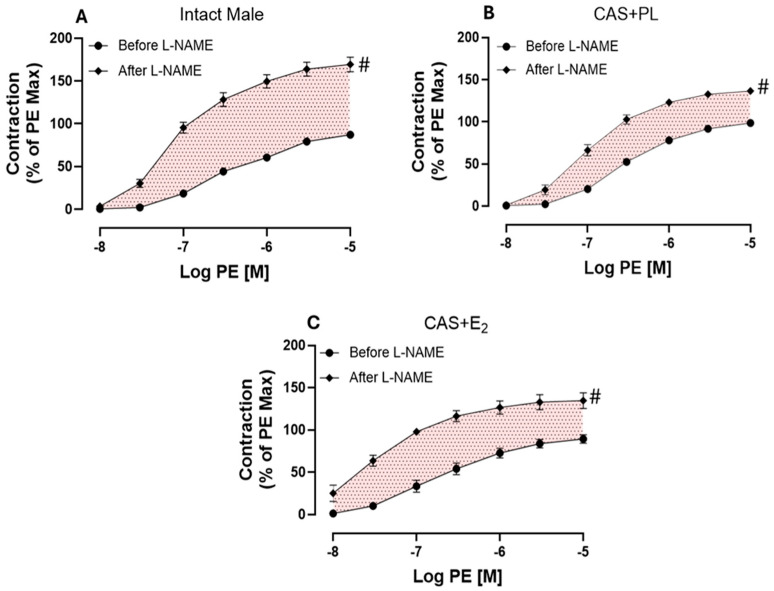
L-NAME potentiation of phenylephrine (PE)-induced vasoconstriction is reduced in castrated rats. PE-induced vasoconstriction in intact aortic rings of (**A**) intact male, (**B**) castrated placebo (CAS + PL) and (**C**) castrated E_2_-treated (CAS + E_2_) rats. Concentration response curve (CRC) to PE (10^−8^ to 10^−5^ M) was generated before and after L-NAME (200 µM) in the presence of Indomethacin (Indo, 10 µM). Results are presented as a percentage of the maximal response to PE obtained in the absence of inhibitor. Data is expressed as mean ± SEM. n = 8 per group, ^#^ *p* < 0.05 vs. before L-NAME, analyzed using two-way ANOVA with repeated measure followed by Šídák’s post hoc test. The shaded area represents the contribution of endothelium-derived nitric oxide (NO) during smooth muscle contraction. L-NAME, Nω-nitro-L-arginine methyl ester.

Next, we assessed endothelium-derived NO during smooth muscle contraction, an indirect measurement of basal NO, using established protocols in the experimental rat groups [10,29,30,32]. Briefly, the CRC to PE was generated in aortic rings before and after pretreatment with L-NAME (200 µM), in the presence of Indo (10 µM). Although addition of Indo had no apparent effects on the contractile response to PE (Supplementary Figure S2), a significant increase in aortic contraction was observed in all three experimental groups after addition of L-NAME (Figure 8A–C).

As shown on Table 7, a significant potentiation was observed in both R_max_ and T_max_ after addition of L-NAME compared to before L-NAME in all groups of animals. However, compared to intact males, the extent of potentiation to PE was significantly lower in aorta of CAS + PL and CAS + E_2_ rats. Although, the ΔAUC did not reach to a significant level in CAS + E_2_ group when compared to the intact males (Table 7).

### 2.8. Effects of E_2_ Treatment on the Regulation of Vascular Signaling Pathways Involved in Aortic Reactivity

**Figure 9:**
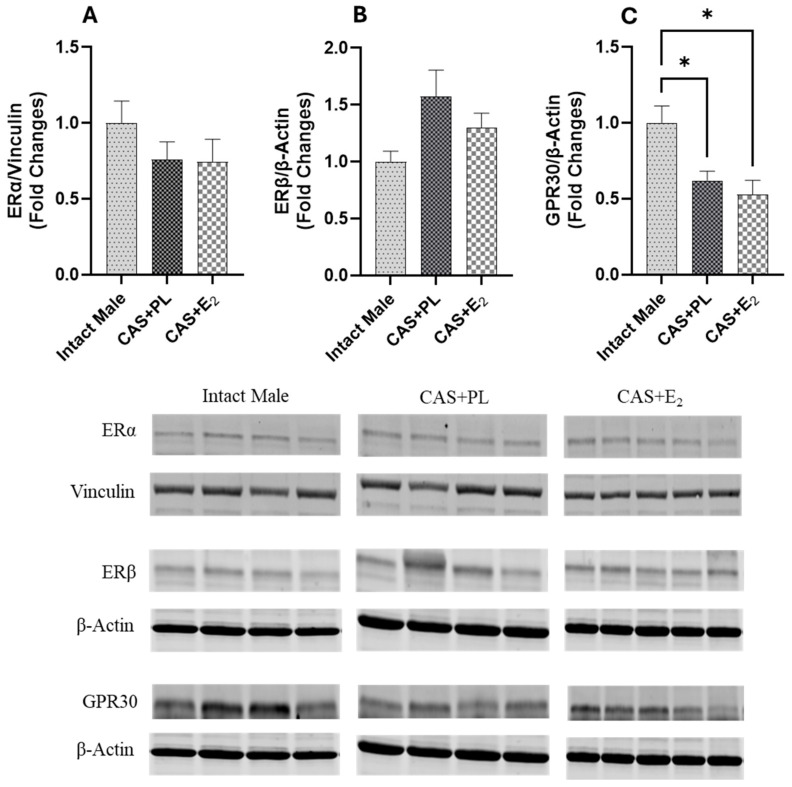
GPR30 expression is reduced in aorta of castrated rats. Western blot analysis of (**A**) ERα, (**B**) ERβ and (**C**) GPR30 in aorta of intact male (n = 4), castrated placebo (CAS + PL, n = 4) and castrated E_2_-treated (CAS + E_2_, n = 5) rats. Protein levels were quantified by densitometric analysis and normalized to corresponding housekeeping protein. Data is expressed as mean ± SEM. Each lane represents a sample from a different rat. Bands of target and housekeeping proteins were shown from the same membrane. Capped lines indicate significant differences between two groups. * *p* < 0.05, analyzed using one-way ANOVA followed by Tukey’s post hoc test. ERα, estrogen receptor α; ERβ, estrogen receptor β; GPR30, G protein-coupled estrogen receptor 30.

**Figure 10:**
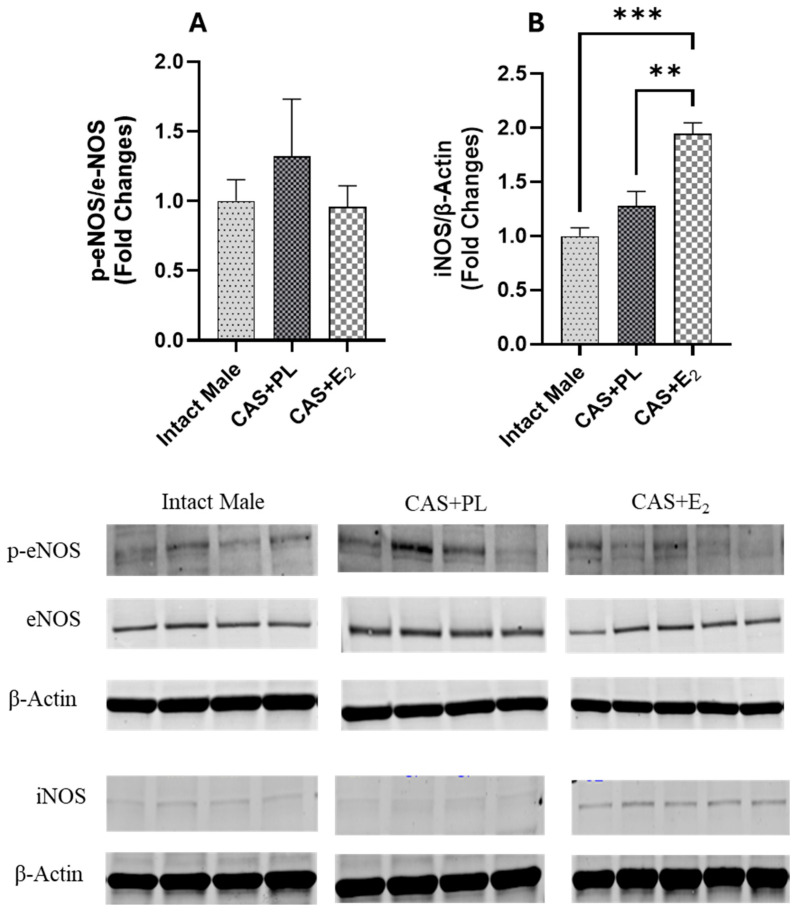
iNOS expression is elevated in aorta of CAS + E_2_ rats. Western blot analysis of (**A**) p-eNOS/eNOS and (**B**) iNOS in aorta of intact male (n = 4), castrated placebo (CAS + PL, n = 4) and castrated E_2_-treated (CAS + E_2_, n = 5) rats. Protein levels were quantified by densitometric analysis and normalized to corresponding housekeeping protein. Data is expressed as mean ± SEM. Each lane represents a sample from a different rat. Bands of target and housekeeping proteins were shown from the same membrane. Capped lines indicate significant differences between two groups. ** *p* < 0.01, *** *p* < 0.001, analyzed using one-way ANOVA followed by Tukey’s post hoc test. eNOS, endothelial nitric oxide synthase; p-eNOS, phosphorylated eNOS; iNOS, inducible nitric oxide synthase.

Western blot analysis of aortic tissue revealed that neither castration nor E_2_ treatment affected the expression levels of ERα and ERβ (Figure 9A,B). In contrast, castration significantly decreased GPR30 expression in the aorta, and E_2_ treatment did not restore it to the level observed in intact male rats (Figure 9C). Additionally, no difference in eNOS activity, as determined by the p-eNOS/eNOS ratio, was detected among the three groups of rats (Figure 10A).

Since iNOS has been shown to be upregulated in vascular injury and inflammation [33,34,35], next, we measured the expression of this protein in aorta of the experimental groups. Although iNOS expression in the aorta was unchanged in castrated rats (CAS + PL) rats, E_2_ treatment in CAS rats resulted in an increase in iNOS expression in the aorta (Figure 10B).

## 3. Discussion

This study demonstrates that E_2_ treatment in castrated male rats improves metabolic parameters but negatively impacts vascular function.

In the current study, our data shows that castration did not affect body weight and vWAT weight, as no differences in these parameters were observed between CAS + PL and intact male rats (Table 2). Similarly, previous studies reported body weight and vWAT weight were unchanged in approximately 25-week-old castrated male Sprague Dawley (SD) rats and 14-week-old castrated male Wistar rats compared to the controls [36,37]. Interestingly, in the present study, E_2_ treatment significantly reduced body weight in castrated rats (Table 2). Consistent with our findings, decreased body weight in E_2_-treated intact as well as castrated male SD rats has also been reported by Brewster et al. and Södersten et al. [38,39]. Furthermore, compared to intact male and CAS + PL groups, the CAS + E_2_ rats showed a reduction in vWAT weight, although adiposity (vWAT weight as a percentage of body weight) remained unchanged (Table 2). This observation aligns with previous research in estrogen-treated C57Bl/6 male mice, which reported decreased body weight without a corresponding change in adiposity [40].

Estrogen has also been implicated in reducing muscle weight; for example, Ikeda et al. demonstrated decrease in both soleus and gastrocnemius muscles in estrogen-treated male mice [41]. It is noteworthy that in the current study, we did not assess other fat depots and muscle weight in the three experimental groups. Therefore, the contribution of other fat depots and skeletal muscle to the observed E_2_-induced reduction in body weight and/or fat weight in castrated male rats cannot be ruled out. A recent study by Iakovleva et al. [42] in sham-operated, castrated and estrogen-treated castrated male C57BL/6J mice reported no significant difference in blood glucose or plasma insulin levels among the groups. Consistently, in our study, we did not observe any significant changes in blood glucose or plasma insulin levels among the groups. However, HbA1c level, while remaining within the normal range, was significantly lower in CAS + E_2_ rats compared to intact male and CAS + PL groups (Table 2). Notably, we also observed improved glucose tolerance in CAS + E_2_ rats at 30, 60 and 90 min following intraperitoneal glucose injection compared to CAS + PL rats (Figure 1A). These findings suggest that E_2_ treatment may enhance glucose handling in castrated male rats. When considering the overall pattern, intact male rats exhibited intermediate glucose handling, which was impaired by castration but partially or fully restored by E_2_ treatment. Although Iakovleva et al. did not assess HbA1c level or perform GTT [42], our findings provide additional insight into the effects of CSHT on long-term glycemic control. It is important to note that HbA1c level reflects average blood glucose level over the preceding 2–3 months, and our animals were 13–15 weeks old at the time of sacrifice. Given the relatively short time frame, the observed difference in HbA1c level, though statistically significant, may not represent a robust biological effect. Nevertheless, when considered alongside the improved glucose tolerance observed in the CAS + E_2_ group, these findings support a modest yet potentially meaningful impact of E_2_ on glucose metabolism in this model. 

Estrogen treatment has been shown to enhance insulin-mediated glycemic control by activating the ERα-Akt-Glut-4 signaling pathway in WAT in both rodents and humans [14]. Specifically, estrogen promotes the translocation of ERα to the plasma membrane, which in turn activates Akt phosphorylation (p-Akt) in a manner similar to insulin signaling [43]. Activated Akt is known to facilitate the translocation of Glut-4 to the plasma membrane, thereby increasing glucose uptake in peripheral tissues [14]. In the present study, we observed increased expression of ERα, p-Akt/Akt ratio, and Glut-4 in ScWAT of CAS + E_2_ rats compared to the CAS + PL group (Figure 2 and Figure 3A, B). These findings suggest that the improvement in glucose homeostasis observed in CAS + E_2_ rats may be partly mediated by activation of the ERα-p-Akt-Glut-4 signaling axis in ScWAT. Because circulating insulin levels were comparable across groups, the metabolic changes in adipose tissue likely reflect direct effects of E_2_–ERα signaling rather than insulin-mediated effects. However, it is important to note that we did not assess Glut-4 translocation to the plasma membrane which is a key step for glucose uptake and a critical indicator of Glut-4 functionality. Furthermore, in our study, we analyzed different adipose depots for morphometric (vWAT) versus molecular (ScWAT) assessments. Since visceral and subcutaneous adipose tissues exhibit distinct metabolic profiles, inflammatory responses, and hormonal sensitivities, the ERα, Akt, and Glut-4 expression changes observed in ScWAT may not fully reflect the mechanisms underlying the beneficial metabolic effects and vWAT weight reduction. While both depots generally respond similarly to estrogen treatment, depot-specific differences in receptor expression and insulin sensitivity could influence the magnitude of these responses.

Prior studies have demonstrated that both ERα and ERβ are expressed in insulin-sensitive tissues, with ERα being more predominant in adipose tissue and implicated in metabolic regulation [44,45]. By contrast, ERβ has been associated with metabolic control but may promote a diabetogenic phenotype, as ERβ deficiency has been shown to protect against diet-induced insulin resistance and glucose intolerance [15,16]. In the current study, however, the assessment of ERβ expression was inconclusive (Supplementary Figure S1), underscoring the need for further investigation into its role in adipose tissue and metabolic homeostasis.

In the current study, although the plasma HDL level was significantly higher in CAS + E_2_ rats compared to intact male and CAS + PL groups, no difference was observed in plasma LDL level among the groups (Table 2). However, CAS + E_2_ rats exhibited a marked elevation in plasma triglyceride level. This pattern is consistent with several clinical studies reporting increased HDL [46,47,48,49] and triglyceride levels [3,8,47] in MtF individuals undergoing estrogen therapy, with or without gender confirming surgery. While the present study did not investigate the mechanism underlying the estrogen-induced rise in triglyceride level, previous work by Dashti et al. suggested that estrogen enhances hepatic fatty acid and triglyceride synthesis and promotes their secretion into the circulation [50]. Similarly, Weinstein et al. demonstrated that estrogen stimulates the release of triglyceride and very low-density lipoprotein (VLDL) from isolated perfused female rat liver [51]. Notably, elevated plasma triglyceride has been reported to impair vascular function [52]. Studies have shown that hypertriglyceridemia can reduce NO bioavailability and impair EDV, contributing to vascular stiffness and endothelial dysfunction [53,54]. Hence, the estrogen-induced increase in circulating triglyceride level could be a contributing factor to the observed vascular dysfunction in CAS + E_2_ rats and may help explain the elevated CVD risk reported in MtF individuals receiving estrogen therapy.

Endothelial dysfunction is widely recognized as a critical early event in the development of vascular dysfunction and CVD. In male rat vascular beds, estrogen treatment has been found to affect the production of PGI_2_ [55,56] and NO [57,58], the two major EDRF involved in aortic relaxation [59,60]. In the present study, the contribution of PGI_2_ in aortic relaxation was assessed using Indomethacin (a COX inhibitor). As shown in Figure 5, ACh-induced relaxation was slightly reduced following Indomethacin administration in all three groups, indicating a minor role for vasorelaxant COX metabolites such as PGI_2_. This finding suggests that COX metabolites are unlikely to be responsible for the vascular impairment observed in CAS + E_2_ rats. Interestingly, both increased and decreased contributions of PGI_2_ have been reported in estrogen-treated male rat aortas. Elevated PGI_2_ were observed in estrogen-treated male SD and Wistar rat aortas in studies conducted by Myers et al. and Wakasugi et al. [55,56], whereas another study reported reduced PGI_2_-induced aortic relaxation in estrogen-treated male SD rats [61]. Notably, those studies used gonadally intact animals, whereas the current study was conducted in castrated rats, which may account for the discrepancy in findings.

The present study demonstrated that E_2_ treatment in castrated male rats not only elevated plasma triglyceride level but also significantly reduced ACh-induced EDV in the aorta, as evidenced by diminished aortic relaxation responses and decreased R_max_ and pD_2_ values to ACh stimulation compared to both the intact male and CAS + PL groups (Figure 4, Table 3). NO is recognized as a key mediator of EDV in large arteries [10,21,22,29]. In the current study, the relative contribution of NO to aortic relaxation was significantly reduced in CAS + E_2_ group compared to both CAS + PL and intact male rats (Figure 5, Table 4). Supporting this observation, a recent study on isolated human arterioles showed that although NO-mediated vasodilation is generally preserved across the lifespan, prolonged (16–20 h) exposure to exogenous estrogen may induce vascular endothelial dysfunction in females younger and older than 40 years [25]. Notably, the adverse effects of estrogen were more pronounced in biological males, who exhibited dysfunction in both endothelial and smooth muscle cells [25]. Together, these findings from SenthilKumar et al. [25] and our study suggest that chronic exposure to exogenous estrogen may predispose blood vessels, particularly in males, to impaired NO-mediated vasorelaxation.

In addition to a potentially altered contribution of NO, the impaired EDV in response to ACh observed in the aortas of CAS + E_2_ rats could be attributed to changes in vascular smooth muscle cell (VSMC) responsiveness to NO or to contractile agents. However, no differences were found in SNP-induced relaxation in the endothelium denuded aortic rings of the experimental groups (Figure 6, Table 5). This finding excludes reduced VSMC sensitivity to NO as the underlying cause of the diminished relaxation observed in CAS + E_2_ rats. Cignarella et al. reported that the vasorelaxation to SNP was attenuated in castrated, estrogen-treated castrated, and estrogen-treated intact male SD rats compared to the controls [61]. Conversely, another study reported enhanced SNP-induced relaxation in endothelium-denuded mesenteric arteries of estrogen-treated male SD rats [62]. This conflicting data could be attributed to differences in experimental design, including the presence or absence of endothelium, vascular bed examined, age and gonad status of the animals, route of administration, as well as the dose and duration of estrogen treatment.

In the present study, the maximum tension (T_max_) to PE was significantly elevated in aortic rings of CAS + E_2_ rats compared with intact males (Figure 7, Table 6). The increased contractility may partially explain the reduced ACh-induced relaxation in the CAS + E_2_ group. Furthermore, L-NAME treatement led to a significantly smaller potentiation of the PE-induced contraction (R_max_) in aortic rings from both CAS + E_2_ and CAS + PL rats (Table 7), suggesting reduced basal NO production during VSMC contraction in castrated animals. However, a significant reduction in ∆AUC was observed only in CAS + PL group, indicating that castration alone may diminish basal NO level (Table 7). Altogether, the decreased aortic relaxation in CAS + E_2_ rats may partly be explained by the elevated aortic contractile response in this group. Although the vasoprotective effects of estrogen in females are well documented [25,63], studies investigating sex-specific vascular effects of estrogen have yielded inconsistent results. For instance, Freay et al. reported no sex differences in estrogen-mediated vasorelaxation in male and female SD rats [64], whereas Hügel et al. observed more pronounced estrogen-mediated vasorelaxation in female Wistar rats [65]. It is important to note that both previous studies were conducted in gonad-intact animals, whereas the present study was performed in castrated male rats.

Estrogen-mediated activation of ERα, ERβ and GPR30 has been proposed to promote vasorelaxation by stimulating eNOS via both genomic and nongenomic pathways [17,66]. ERα has been shown to protect against vascular endothelial cell injury, while ERβ is implicated in the regulation of arterial tone and blood pressure [67]. In contrast, the loss of GPR30 has been associated with enhanced endothelium-dependent vasoconstriction, accelerated atheroma formation, and increased inflammation [68]. In the current study, Western blot analysis revealed no significant differences in aortic ERα and ERβ expression among the experimental groups (Figure 9A, B), suggesting that altered expression of these receptors is unlikely to be involved in the reduced aortic relaxation observed in CAS + E_2_ rats. However, GPR30 expression was significantly lower in both CAS + PL and CAS + E_2_ rats compared to intact males (Figure 9C). Supporting this observation, Lindsey et al. reported diminished estrogen-induced mesenteric arterial relaxation in male Lewis rats, attributing the effect to downregulation of GPR30 [69]. Similarly, studies in estrogen-treated ER-positive MCF-7 breast cancer cells have demonstrated downregulation of both ERα and GPR30 [70]. Emerging evidence indicates that the expression pattern of estrogen receptor isoforms vary across tissues and species, contributing to functional heterogeneity [71]. However, this area of research is still evolving and has yet to fully elucidate how such receptor variability influences estrogen’s effects on specific organ systems, such as the vasculature.

Finally, it is important to note that L-NAME used in the current study is a non-selective NOS inhibitor; therefore, we assessed the expression of both eNOS and iNOS to identify which isoform might contribute to the impaired EDV observed in CAS + E_2_ rats. Our data indicates that the eNOS activity, as reflected by the p-eNOS/eNOS ratio, was not altered by E_2_ treatment in the aorta of castrated rats (Figure 10A). This finding aligns with study by Sobey et al. who reported no changes in eNOS expression in the carotid arteries of estrogen- or vehicle-treated SD male rats [72]. However, they reported elevated plasma NO levels in estrogen-treated rats, which were attributed to decreased expression of caveolin-1 and increased calmodulin expression, both key modulators of eNOS activity [72]. Although we did not assess the plasma NO level or these regulatory proteins, the increased iNOS expression observed in the aorta of CAS + E_2_ rats (Figure 10B) may indicate a potential involvement of iNOS in the vascular impairment seen in this group. The differential regulation of eNOS and iNOS has previously been reported to be associated with estrogen dose. For instance, in cultured human umbilical vascular endothelial cells (HUVECs), treatment with 1 mM estrogen reduced eNOS mRNA and increased iNOS mRNA level compared to treatment with 1 nM estrogen. This shift was attributed to increased cell permeability induced by high estrogen concentrations [73]. Similarly, high-dose estrogen has been shown to increase iNOS expression in VSMC [74]. In our study, we analyzed intact aortic tissue without separating the endothelium from the smooth muscle layer, and thus the source of increased iNOS remains undetermined. Interestingly, a link between GPR30 and iNOS expression has been reported in cardiac tissue. Wang et al. reported that activation of GPR30 downregulates iNOS expression, reduces apoptosis, and inhibits cardiac fibroblast proliferation, suggesting a protective role for GPR30 in limiting myocardial fibrosis [75]. In our study, we noted reduced GPR30 and elevated iNOS expression in the aortas of CAS + E_2_ rats. However, these observations do not establish a mechanistic link, and further studies are needed to determine whether these pathways mediate the effects of E_2_ on aortic relaxation.

In conclusion, this study highlights the metabolic and vascular effects of E_2_ treatment in CAS male rats. Findings from our CSHT rat model suggest that E_2_ treatment reduced body weight and improved HbA1c level in CAS rats, along with enhanced glucose tolerance compared to the CAS + PL group. These metabolic improvements were associated with upregulation of ERα, p-Akt/Akt, and Glut-4 expression in ScWAT. However, E_2_ also increased plasma triglyceride level and impaired EDV, indicating compromised vascular function. Our results suggest that impaired aortic relaxation in the CAS + E_2_ group may be partly attributable to increased contractility. In addition, we observed reduced GPR30 expression and elevated iNOS expression. Further studies are required to elucidate the mechanisms by which E_2_ modulates aortic relaxation in castrated male rats, particularly the potential link between GPR30 and NO signaling. While species differences must be acknowledged, these findings offer preliminary insights into cardiovascular pathophysiology in MtF individuals undergoing CSHT and may hold potential translational relevance with further validation.

Limitation: We acknowledge several limitations in the current study. The relatively small sample sizes for the Western blot analyses may have limited our ability to detect modest changes in protein expression; thus, future studies with larger cohorts will be important to strengthen these molecular findings. Another limitation of our study design is the absence of an Intact Male + E_2_ group, which would have further clarified whether the observed changes in the pAkt–Glut-4 pathway were directly attributable to E_2_ administration. Additionally, the relatively short intervention period limits the generalizability of our findings and precludes conclusion about long-term effects. Furthermore, while morphometric analyses were conducted on vWAT, protein expression studies were performed on ScWAT. To establish more direct mechanistic links, future investigations should assess protein expression within the same adipose depot used for morphometric analysis. Building on these findings, our upcoming studies aim to expand mechanistic insight through assessments such as glucose uptake in WAT and a broader spectrum of cardiometabolic endpoints.

## 4. Materials and Methods

### 4.1. Chemicals

All Chemicals were purchased from Sigma Aldrich (St. Louis, MO, USA) and dissolved in water unless indicated otherwise.

### 4.2. Experimental Animals

8-week-old castrated (CAS) male Sprague Dawley (SD) and age-matched intact male rats were purchased from Charles River Laboratories (Wilmington, MA, USA) and housed in the animal facility at the University of the Pacific. After 1–2 weeks of acclimatization, the CAS rats were subcutaneously implanted either with placebo (PL) or 1.5 mg 17β-estradiol (E_2_) (60 days uniform release) pellets (Innovative Research of America, Sarasota, FL, USA).

The rats were housed in a vivarium with controlled humidity and temperature at the University of the Pacific. They were maintained on a 12 h light/dark cycle and provided with ad libitum access to water and standard rodent chow (Mazuri rodent chow). All animal study related protocols were approved by the Animal Care Committee at the University of the Pacific and complied with the Guide for the Care and Use of Laboratory Animals: Eighth Edition [Institutional Animal Care and Use Committee (IACUC, 21R01). The animals were euthanized using carbon dioxide (CO_2_) after ~35 days of the implantation of the pellets (aged 13–15 weeks), in accordance with the procedures recommended in the 2013 AVMA Guidelines on Euthanasia and the NIH Guidelines for the Care and Use of Laboratory Animals.

The intra-abdominal visceral white adipose tissue (vWAT) was collected from both the mesenteric depot (surrounding the small intestine and its vasculature) and the omental depot (greater omentum), following the careful removal of the mesenteric arterial cascade. Both depots were combined for total vWAT weight measurements. The ratio of vWAT weight to total body weight was then calculated, with the results expressed as the percentage of vWAT weight relative to the total body weight.

### 4.3. Measurement of Metabolic Parameters in Blood and Plasma

The glucose tolerance test (GTT) was performed before sacrificing the animals. Briefly, the rats were fasted overnight. Next morning, blood was collected via a tail nick to assess the fasting blood glucose using a standard glucometer (OneTouch UltraMin). Glucose solution (2 g/Kg body weight) was then injected intraperitoneally, and blood glucose level was measured at 15, 30, 45, 60, 90, and 120 min.

At the time of sacrifice, blood obtained by intra-cardiac puncture was used to measure non-fasting blood glucose using OneTouch UltraMin, and glycated hemoglobin (HbA1c) level using A1cNow kit (PTS diagnostics, Sunnyvale, CA, USA). Additionally, for collection of plasma, blood was taken in tubes containing anticoagulants (heparin and sodium citrate), centrifuged at 10,000× *g* for 5 min at 4 °C, aliquoted and stored at −80 °C for further analysis. Plasma insulin (#10-1250-01, Mercodia, Uppsala, Sweden), E_2_ (#ab108667, Abcam, Waltham, MA, USA), testosterone (#ab285350, Abcam, Waltham, MA, USA), HDL (#79970, Crystal Chem, Elk Grove Village, IL, USA), LDL (#79960, Crystal Chem, Elk Grove Village, IL, USA) and triglyceride (#TR22421, Thermo Fisher Scientific, Waltham, MA, USA) levels were measured using kits according to the manufacturer’s protocols.

### 4.4. Measurement of Aortic Tension

The thoracic aortas were excised, cleaned of adhering connective tissue, and cut into 2 mm rings. Isometric tension was measured by suspending the rings horizontally between two stainless steel hooks in separate organ baths, each containing 20 mL of Krebs buffer (in mM: 119 NaCl, 4.7 KCl, 1.18 KH_2_PO_4_, 1.17 MgSO_4_, 24.9 NaHCO_3_, 0.023 EDTA, 1.6 CaCl_2_, and 6.0 glucose) at 37 °C. The organ baths were continuously bubbled with 95% O_2_ and 5% CO_2_. Isometric tension was monitored in real-time using a computer-based data acquisition system (PowerLab; ADInstruments, Colorado Springs, CO, USA). The aortic rings were equilibrated under a resting tension of 1 g for 40 min to achieve a stable basal tone. The rings were subsequently stimulated twice with 80 mM KCl at 20 min intervals until maximum contraction was reached. A submaximal concentration of PE (2 μM) that elicited 80% of the maximal response (EC_80_) was used for relaxation studies [10]. The ability of acetylcholine (ACh, 10 μM) to induce relaxation in phenylephrine (PE, 2 μM)-pre-contracted vessels served as an indicator of intact endothelial function.

#### 4.4.1. Measurement of Aortic Relaxation

EDV was measured in PE (2 µM) precontracted aortas. The ACh concentrations used to generate the relaxation curves were determined based on the standard protocol previously reported by our group [10,29,76]. Briefly, PE precontracted aortic rings were used to generate the concentration-response curves (CRC) to ACh (10^−8^ to 10^−5^ M) or sodium nitroprusside (SNP, 10^−9^ to 10^−5^ M), a NO donor. After the first CRC was generated, the aortic rings were washed three times with Krebs solution to restore them to their basal tone. Next, a non-selective Cyclooxygenase (COX) inhibitor Indomethacin (Indo, 10 μM, dissolved in DMSO) was added in the organ bath. The aortic tissue was incubated for 20 min and the second CRC to ACh was generated in the precontracted ring. Following the same procedures, the third CRC to ACh was obtained after incubating the aortic ring with combination of Indo and Nω-nitro-L-arginine methyl ester (L-NAME, 200 µM), a nonselective nitric oxide synthase (NOS) inhibitor for 20 min. Since the addition of L-NAME in this study completely blocked the aortic relaxation to ACh, the relaxation was recorded as zero.

#### 4.4.2. Measurement of Aortic Contraction

First the CRC to PE (10^−8^ to 10^−5^ M) was generated in absence of any inhibitors. The aortic rings were then incubated for 20 min with Indo (10 μM) before the generation of second CRC. Lastly, for the third CRC to PE, the aortic rings were treated with Indo (10 μM) followed by L-NAME (200 μM) for 20 min. Because the effect of the COX inhibitor on the CRC to PE was minimal and negligible, only CRCs before and after addition of L-NAME are shown in Figure 8. The effect of Indo is shown in Supplementary Figure S2.

### 4.5. Western Blot Analysis

The harvested tissue samples such as subcutaneous white adipose tissue (ScWAT) and aorta collected after euthanizing the animals were flash-frozen by liquid nitrogen and saved at −80 °C for later analysis. We used T-PER tissue dissociation buffer (#78510, Thermo Fisher Scientific, Waltham, MA, USA) supplemented with phosphatase and protease inhibitor cocktail for protein extraction from the tissues. First, tissues were taken in M-tubes (#130096335, Miltenyi Biotech, Bergisch, Germany) containing the buffer cocktail and then the tubes were placed on the gentleMACS Dissociator (Miltenyi Biotech, Bergisch, Germany). Next, the protein extraction protocol was selected from the menu and after 1 min, the blended tissue was centrifuged at 15,000× *g* at 4 °C for 15 min. After centrifugation, the supernatants were collected, and BCA gold assay (#A55861, Thermo Fisher Scientific, Waltham, MA, USA) was performed to determine the total protein concentration of the samples. 30 μg (aorta) or 50 μg (adipose) of protein samples were loaded in the sodium dodecyl sulfate polyacrylamide gel electrophoresis (SDS-PAGE) wells (Bio-Rad Laboratories, CA, USA). 0.45 μm nitrocellulose membrane (#1620115, Bio-Rad Laboratories, Hercules, CA, USA) and Transblot Turbo Transfer System (Bio-Rad Laboratories, Hercules, CA, USA) were used for protein transfer. Next, the membranes were blocked for 1 h with commercially available blocking buffer (#927-60001, LI-COR Biosciences, Lincoln, NE, USA).

The host species for all the primary antibodies was rabbits. Antibodies were diluted with commercially available antibody diluent (#927-65001, LI-COR Biosciences, Lincoln, NE, USA) and the membranes were incubated with the primary antibodies overnight at 4 °C. Primary antibodies for Akt (#9272), p-Akt (Ser473) (#4060), eNOS (#32027), p-eNOS (Ser1177) (#9571), vinculin (#4650), β-Actin (#4970) and GAPDH (#2118) were purchased from Cell Signaling Technology (Danvers, MA, USA). Antibodies against ERα (#ab32063), ERβ (#ab3576) and Glut-4 (#ab654) were obtained from Abcam (Waltham, MA, USA) and antibodies against GPR30 (#PA5-28647) and iNOS (#PA1-036) were purchased from Thermo Fisher Scientific (Waltham, MA, USA). 1:1000 dilution was used for all primary antibodies except ERα (1:500), ERβ (1:300), GPR30 (1:500), p-Akt (1:500), p-eNOS (1:300), β-Actin (1:4000) and GAPDH (1:10,000). Evaluation of targets (Akt and p-Akt and eNOS and p-eNOS) was performed on the same gel and membrane to ensure accurate detection and measurement of phosphorylated and total protein. After incubation with primary antibody, the membranes were washed 3–4 times with TBS containing 0.1% Tween-20 and incubated for 1 h at room temperature with IRDye 680 Donkey anti-Rabbit IgG secondary antibody (dilution 1:10,000; LI-COR Biosciences, Lincoln, NE, USA). Finally, after removing the secondary antibody, membranes were washed again 3–4 times and bands were detected using the LI-COR Odyssey M imaging system (Serial Number #ODM-0412, LI-COR Biosciences, Lincoln, NE, USA). The bands were quantified by densitometry using LI-COR Image Studio Lite software v5.2 (LI-COR Biosciences, Lincoln, NE, USA). To confirm the uniformity of protein loading, membranes were incubated with vinculin, β-Actin or GAPDH antibodies for the detection of housekeeping proteins. Then, the target protein bands were normalized to the respective housekeeping protein levels and expressed as fold changes compared to the other groups. The full blot images are shown in Supplementary Figures S3 and S4. 

### 4.6. Statistical Analysis

The data was analyzed with GraphPad Prism v10.4.0. (GraphPad Software Inc., La Jolla, CA, USA) and is presented as mean ± standard error of the mean (SEM). A one-way ANOVA was conducted to compare the group means (e.g., metabolic parameters, R_max_, pD_2_, ΔAUC, protein expression etc.), followed by Tukey’s post hoc analysis, with the effect of hormone considered as the independent factor. Tukey’s post hoc test was used for multiple comparisons when comparing all groups to each other (e.g., metabolic parameters, protein expression), as it controls familywise error rate across all possible pairwise comparisons. The GTT and comparison of CRC to agonists (ACh, SNP, or PE) among different groups were analyzed by two-way ANOVA with repeated measure followed by Tukey’s post hoc test. Two variables were groups and agonist concentration/blood glucose. Agonist concentration/blood glucose was considered as the repeated-measure factor.

Relaxation responses to agonists (ACh and SNP) were calculated as the percent (%) relaxation from the maximum PE (2 μM) contraction at each concentration. To calculate the sensitivity to the agonist [pD_2_ (−logEC_50_)], we used a sigmoidal dose–response model with a variable slope to measure the concentration that induced half of the maximum relaxation (EC_50_). The maximum relaxation response to the agonist was referred to as R_max_, and the maximum tension to the contractile agent was termed T_max_.

To compare the ACh CRC before and after inhibitor treatment within each group, a two-way ANOVA with repeated measures, followed by Tukey’s post hoc test was applied. On the other hand, a two-way ANOVA with repeated measures, followed by Šídák’s post hoc test was used for comparison of PE CRC before and after L-NAME treatment in the presence of Indo within each group. The two variables were agonist concentration and pre- and post-inhibitor treatment. The agonist concentration was considered as the repeated-measure factor.

The ΔAUC (AUC, area under the curve) represents the difference between two CRC, comparing the absence and presence of inhibitors. It was measured to determine the relative contribution of different endothelium-derived relaxing factors (EDRF) to ACh-induced vasorelaxation or production of endothelial NO during smooth muscle contraction to PE.

### 4.7. Database and Inclusion/Exclusion Criteria

We used PubMed and Google Scholar for our database search. For inclusion and exclusion criteria, only studies with consistent strain, age, sex, surgical technique, drug, dose, and treatment duration were included, while animals showing signs of illness were excluded. Endothelial function was confirmed by ACh (10 μM)-induced relaxation of PE (2 μM)-precontracted vessels. Overall, explicit attention to randomization and blinding has strengthened the internal validity of the present study.

## Figures and Tables

**Table 1:** Endpoint measurement of plasma testosterone and estrogen levels in intact male, castrated placebo (CAS + PL) and castrated E_2_-treated (CAS + E_2_) rats.

Group	n	Testosterone (ng/mL)	Estrogen (pg/mL)
Intact Male	5	3.35 ± 1.37	1.96 ± 0.33
CAS + PL	5	0.00 ± 0.03 ^a^	2.23 ± 0.40
CAS + E_2_	5	0.03 ± 0.02 ^a^	462.25 ± 70.16 ^ab^

Data is expressed as mean ± SEM. ^a^ *p* < 0.05 vs. intact male, ^b^ *p* < 0.05 vs. CAS + PL, analyzed using one-way ANOVA followed by Tukey’s post hoc test.

**Table 2:** Body weight, visceral white adipose tissue (vWAT) weight, blood glucose, HbA1c, plasma insulin, HDL, LDL and triglyceride levels in intact male, castrated placebo (CAS + PL) and castrated E_2_-treated (CAS + E_2_) rats.

Metabolic Parameter	n	Intact Male	CAS + PL	CAS + E_2_
Body Weight (g)	11	360.00 ± 10.82	381.91 ± 9.56	267.45 ± 6.34 ^ab^
vWAT (g)	9	3.25 ± 0.34	4.13 ± 0.57	1.68 ± 0.14 ^ab^
vWAT/Body Weight (%)	9	0.86 ± 0.09	1.00 ± 0.09	0.80 ± 0.12
Non-Fasting Blood Glucose (mg/dL)	9	109.44 ± 8.93	107.56 ± 8.20	99.11 ± 5.01
HbA1c (%)	8	4.38 ± 0.08	4.31 ± 0.11	4.03 ± 0.03 ^ab^
Insulin (ng/mL)	10	0.80 ± 0.13	0.62 ± 0.07	0.75 ± 0.11
HDL (mg/dL)	10	38.20 ± 2.26	44.25 ± 3.79	66.79 ± 3.11 ^ab^
LDL (mg/dL)	9	13.78 ± 1.50	11.67 ±1.05	14.44 ± 1.53
Triglyceride (mg/dL)	10	106.54 ± 10.28	85.36 ± 5.34	147.91 ± 13.25 ^ab^

Data is expressed as mean ± SEM. ^a^ *p* < 0.05 vs. intact male, ^b^ *p* < 0.05 vs. CAS + PL, analyzed using one-way ANOVA followed by Tukey’s post hoc test.

**Table 3:** Maximum relaxation (R_max_) and sensitivity (pD_2_: −logEC_50_) to acetylcholine (ACh) in intact aortic rings of intact male, castrated placebo (CAS + PL) and castrated E_2_-treated (CAS + E_2_) rats.

Group	n	R_max_ (%)	pD_2_
Intact Male	8	83.02 ± 1.46	7.24 ± 0.10
CAS + PL	8	82.39 ± 2.39	7.11 ± 0.09
CAS + E_2_	8	67.94 ± 1.59 ^ab^	6.62 ± 0.11 ^ab^

Data is expressed as mean ± SEM. ^a^ *p* < 0.05 vs. intact male, ^b^ *p* < 0.05 vs. CAS + PL, analyzed using one-way ANOVA followed by Tukey’s post hoc test.

**Table 4:** A comparison among area under the curve (∆AUC), maximum relaxation (R_max_) and sensitivity (pD_2_: −logEC_50_) to acetylcholine (ACh) without inhibitor, after Indo, and after Indo + L-NAME in intact aortic rings of intact male, castrated placebo (CAS + PL) and castrated E_2_-treated (CAS + E_2_) rats.

Group	No Inhibitor			After Indo			After Indo + L-NAME		
	R_max_ (%)	pD_2_	ΔAUC	R_max_ (%)	pD_2_	ΔAUC	R_max_ (%)	pD_2_	ΔAUC
Intact Male	83.56 ± 2.20	7.25 ± 0.14	ND	83.66 ± 1.78	7.03 ± 0.10	17.10 ± 6.70	0.00 *^#^	ND	163.98 ± 1.94 ^#^
CAS + PL	83.28 ± 2.45	7.15 ± 0.10	ND	83.43 ± 2.53	6.99 ± 0.18	13.70 ± 7.09	0.00 *^#^	ND	155.89 ± 8.87 ^#^
CAS + E_2_	69.65 ± 2.68 ^ab^	6.57 ± 0.15 ^ab^	ND	65.18 ± 2.56 ^ab^	6.39 ± 0.14 ^ab^	17.60 ± 6.58	0.00 *^#^	ND	94.30 ± 9.36 ^#ab^

Data is expressed as mean ± SEM. n = 8 per group, * *p* < 0.05 vs. no inhibitor, ^#^ *p* < 0.05 after Indo, analyzed using two-way ANOVA followed by Šídák’s post hoc test. ^a^ *p* < 0.05 vs. intact male, ^b^ *p* < 0.05 vs. CAS + PL, analyzed using one-way ANOVA followed by Tukey’s post hoc test. ND, not determined. Indo, Indomethacin; L-NAME, Nω-nitro-L-arginine methyl ester.

**Table 5:** Maximum relaxation (R_max_) and sensitivity (pD_2_: −logEC_50_) to sodium nitroprusside (SNP) in endothelium denuded aortic rings of intact male, castrated placebo (CAS + PL) and castrated E_2_-treated (CAS + E_2_) rats.

Group	n	R_max_ (%)	pD_2_
Intact Male	8	100.89 ± 0.93	7.87 ± 0.07
CAS + PL	8	100.59 ± 2.01	7.85 ± 0.13
CAS + E_2_	8	100.23 ± 0.91	7.85 ± 0.07

Data is expressed as mean ± SEM, analyzed using one-way ANOVA.

**Table 6:** Maximum tension (T_max_) and sensitivity (pD_2_: −logEC_50_) to phenylephrine (PE) in intact aortic rings of intact male, castrated placebo (CAS + PL) and castrated E_2_-treated (CAS + E_2_) rats.

Group	n	T_max_ (g)	pD_2_
Intact Male	8	1.85 ± 0.14	6.57 ± 0.06
CAS + PL	8	2.51 ± 0.15	6.70 ± 0.08
CAS + E_2_	8	2.75 ± 0.29 ^a^	6.90 ± 0.14

Data is expressed as mean ± SEM. ^a^ *p* < 0.05 vs. intact male, analyzed using one-way ANOVA followed by Tukey’s post hoc test.

**Table 7:** A comparison among area under the curve (∆AUC), maximum relaxation (R_max_), maximum tension (T_max_) and sensitivity (pD_2_: −logEC_50_) to phenylephrine (PE) before and after L-NAME treatment in the presence of Indomethacin in intact aortic rings of intact male, castrated placebo (CAS + PL) and castrated E_2_-treated (CAS + E_2_) rats.

	n	R_max_ (%)	T_max_ (g)	pD_2_	ΔAUC
**Intact Male**	8				
Before L-NAME		87.08 ± 3.24	1.64 ± 0.16	6.47 ± 0.05	-
After L-NAME		169.43 ± 8.66 ^#^	3.11 ± 0.20 ^#^	7.17 ± 0.06	203.72 ± 20.22
**CAS + PL**	8				
Before L-NAME		98.58 ± 2.71	2.44 ± 0.19	6.57 ± 0.04	-
After L-NAME		136.62 ± 3.05 ^#a^	3.36 ± 0.2 ^#^	7.09 ± 0.13	109.66 ± 7.02 ^a^
**CAS + E_2_**	8				
Before L-NAME		89.30 ± 4.98	2.73 ± 0.41 ^a^	6.84 ± 0.17	-
After L-NAME		134.70 ± 9.33 ^#a^	3.83 ± 0.28 ^#^	8.69 ± 0.72 ^#ab^	159.16 ± 28.31

Data is expressed as mean ± SEM. ^#^ *p* < 0.05 vs. before L-NAME, analyzed using two-way ANOVA followed by Šídák’s post hoc test. ^a^ *p* < 0.05 vs. intact male, ^b^ *p* < 0.05 vs. CAS + PL, analyzed using one-way ANOVA followed by Tukey’s post hoc test. L-NAME, Nω-nitro-L-arginine methyl ester.

## Data Availability

The original contributions presented in this study are included in the article/Supplementary Material. Further inquiries can be directed to the corresponding author.

## Supplementary Materials

The following supporting information can be downloaded at: https://www.mdpi.com/article/10.3390/ijms26188885/s1.
