# Supplementary material for: Effects of 17β-Estradiol Treatment on Metabolic Function and Aortic Relaxation in Castrated Male Rats
Source: Int J Mol Sci. 2025 Sep 12;26(18):8885. doi: 10.3390/ijms26188885 (PMC12469970; doi:10.3390/ijms26188885)
Supplement: Supplementary file 1 [file ijms-26-08885-s001.zip › ijms-3837989-supplementary.pdf]

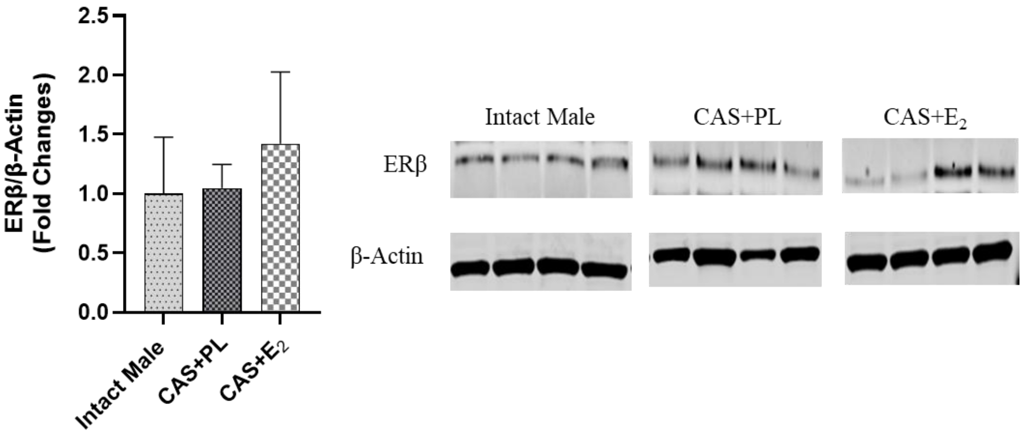

Figure S1. Western Blot analysis of ERβ in subcutaneous white adipose tissue of intact male, castrated placebo- (CAS+PL) and castrated E_2-treated (CAS+E_2) rats. Protein levels were quantified by densitometric analysis and normalized to corresponding housekeeping protein. Data is expressed as mean ± SEM. N = 4 per group. Each lane represents a sample from a different rat. Bands of target and housekeeping proteins were shown from the same membranes, analyzed using one-way ANOVA. ERβ, estrogen receptor β.

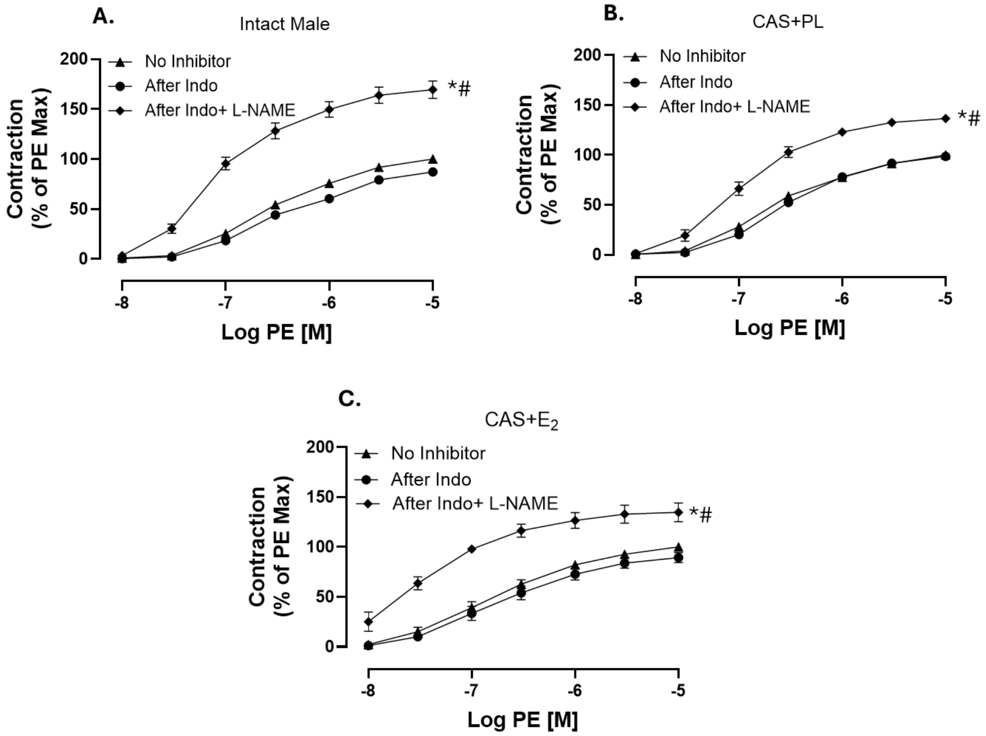

Figure S2. Phenylephrine (PE)-induced vasoconstriction in intact aortic rings of (**A**) intact male, (**B**) castrated placebo- (CAS+PL), and (**C**) castrated E_2-treated (CAS+E_2) rats. Concentration response curve (CRC) to PE (10^{-8} to 10^{-5} M) was generated in absence of inhibitor (No Inhibitor), or in presence of Indomethacin (Indo, 10 μM) followed by addition of L-NAME (200 μM) (Indo + L-NAME). Results are expressed as a percentage of the maximal contraction to PE obtained in the absence of inhibitor. Data is expressed as mean ± SEM. N = 8 per group, $^{*}p<0.05$ vs No Inhibitor, $^{\#}p<0.05$ vs Indo, analyzed using two-way ANOVA with repeated measure followed by Tukey's post hoc test. Indo, Indomethacin; L-NAME, Nω-nitro-L-arginine methyl ester.

A.

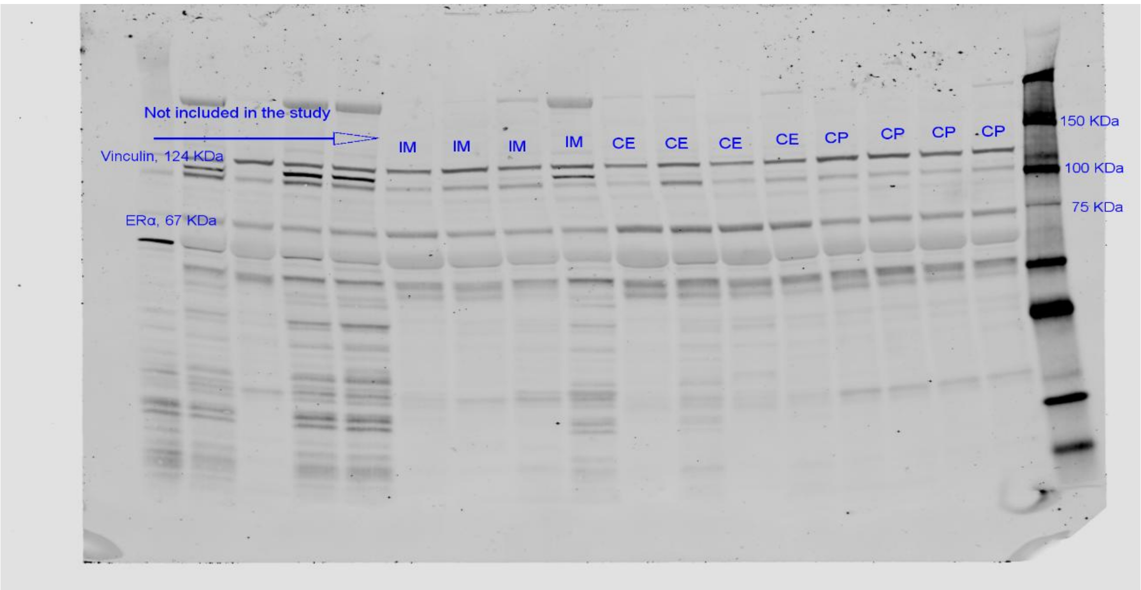
Not included in the study
Vinculin, 124 KDa
ERα, 67 KDa
IM IM IM IM CE CE CE CE CP CP CP CP
150 KDa
100 KDa
75 KDa

B.

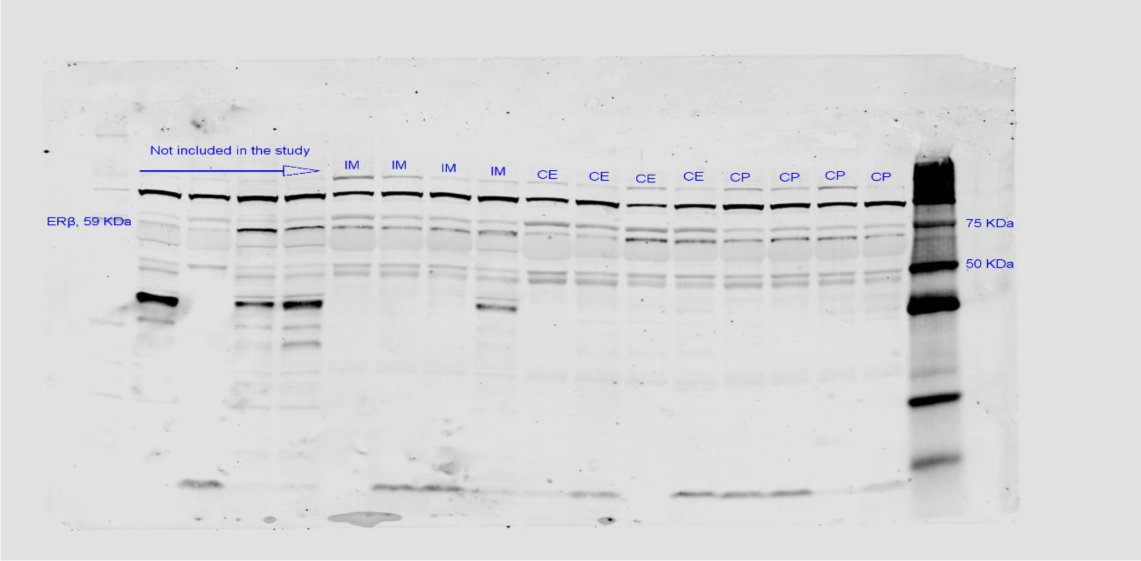
Not included in the study
IM IM IM IM CE CE CE CE CP CP CP CP
ERβ, 59 KDa
75 KDa
50 KDa

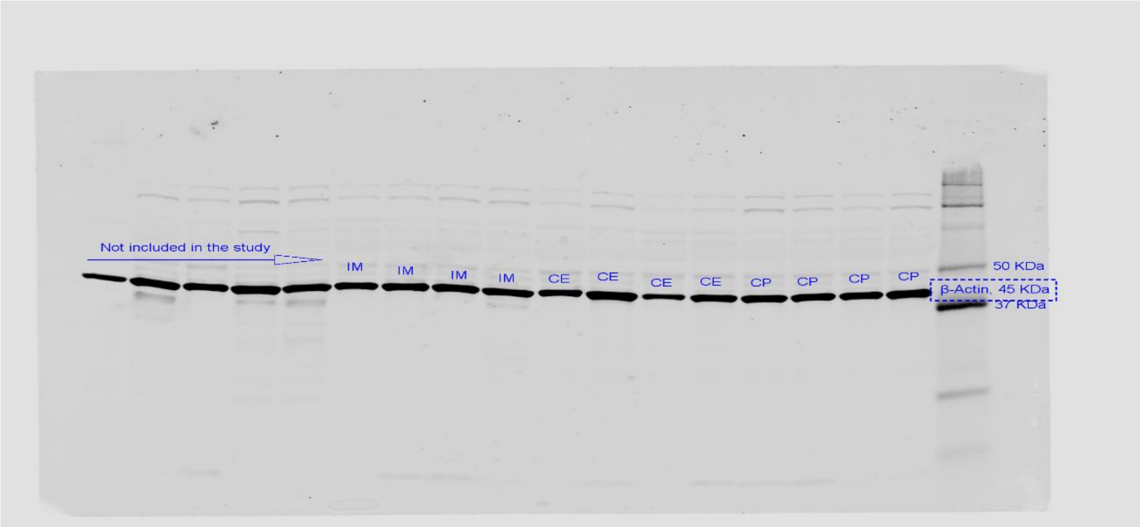
Not included in the study
IM IM IM IM CE CE CE CE CP CP CP CP
50 KDa
β-Actin, 45 KDa
37 KDa

C.

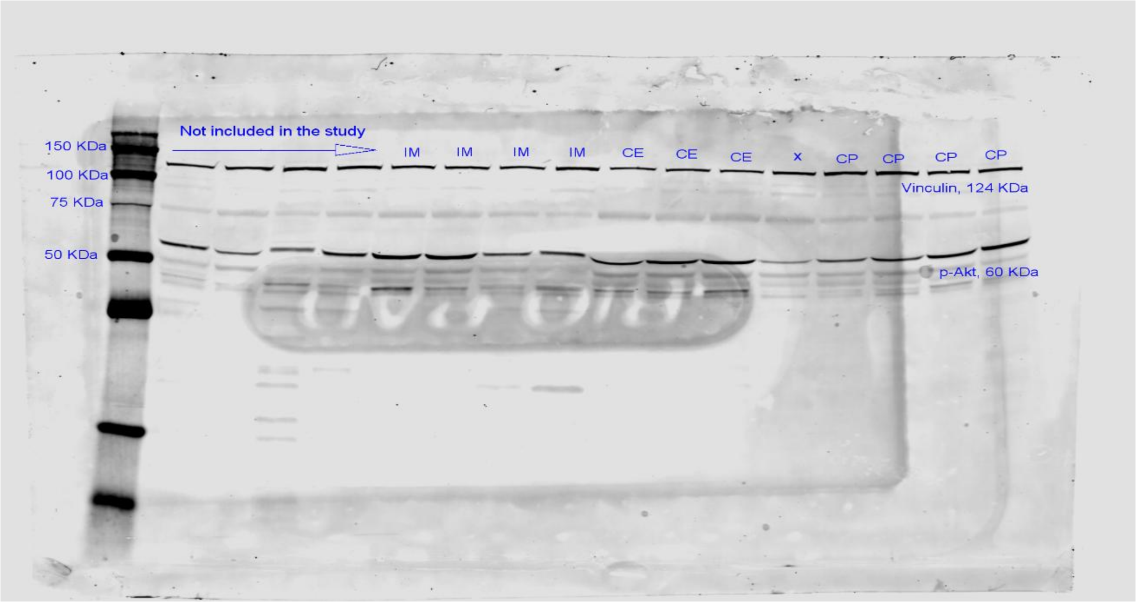
150 KDa
100 KDa
75 KDa
50 KDa
Not included in the study
IM
IM
IM
IM
CE
CE
CE
X
CP
CP
CP
CP
Vinculin, 124 KDa
p-Akt, 60 KDa

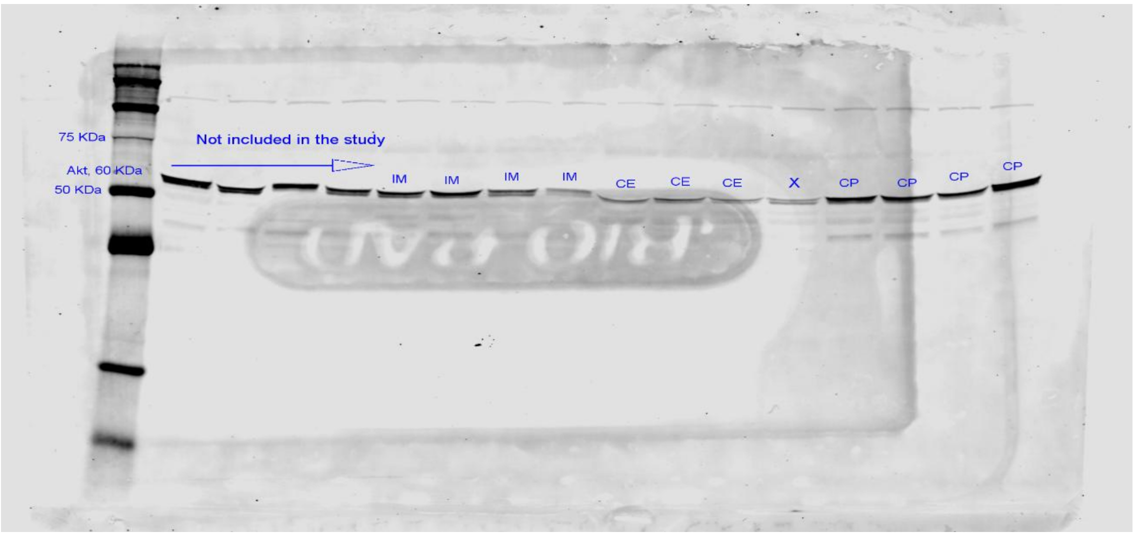
75 KDa
Akt, 60 KDa
50 KDa
Not included in the study
IM
IM
IM
IM
CE
CE
CE
X
CP
CP
CP
CP

D.

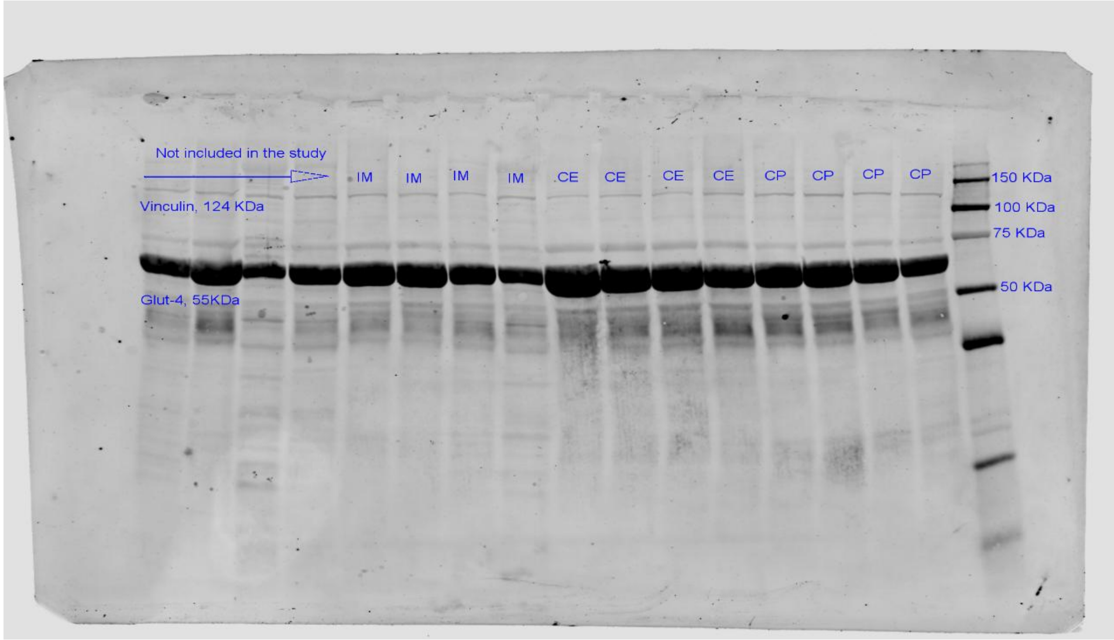

Figure S3. Western blot images of (A) ERα; (B) ERβ; (C) pAkt and Akt; (D) Glut-4 expression in subcutaneous white adipose tissue (ScWAT) of intact male (IM, n=4), castrated placebo (CP, n=4)) and CAS E_2-treated (CE, n=3 for pAkt/Akt and n = 4 for ERα, ERβ and Glut-4) rats. Protein levels were quantified by densitometric analysis and normalized to corresponding housekeeping protein. Each lane represents a sample from a different rat. Bands of target and housekeeping proteins were shown from the same membranes. ERα, estrogen receptor α; ERβ, estrogen receptor β, p-Akt, phosphorylated Akt; Glut-4, glucose transporter-4. Parts of these images were used in **Figures 2 and 3A-B** in the Manuscript and in **Supplementary Figure S1**.

A.

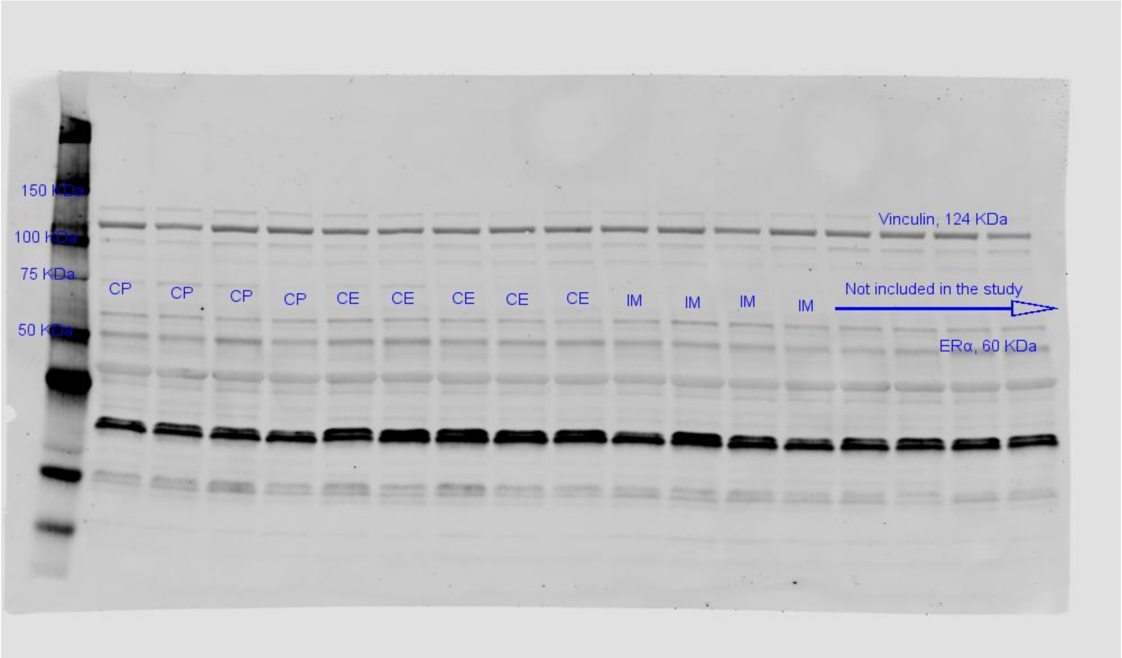
150 KDa
100 KDa
75 KDa
50 KDa
Vinculin, 124 KDa
CP
CP
CP
CP
CE
CE
CE
CE
CE
IM
IM
IM
IM
Not included in the study
ERα, 60 KDa

B.

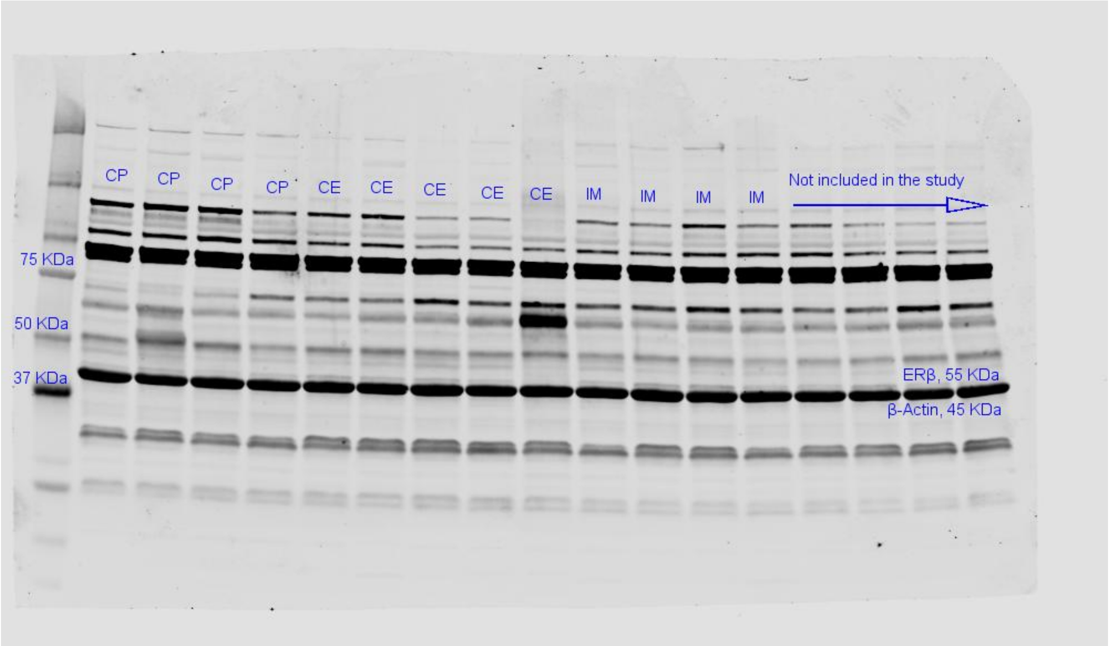
CP
CP
CP
CP
CE
CE
CE
CE
CE
IM
IM
IM
IM
Not included in the study
75 KDa
50 KDa
37 KDa
ERβ, 55 KDa
β-Actin, 45 KDa

C.

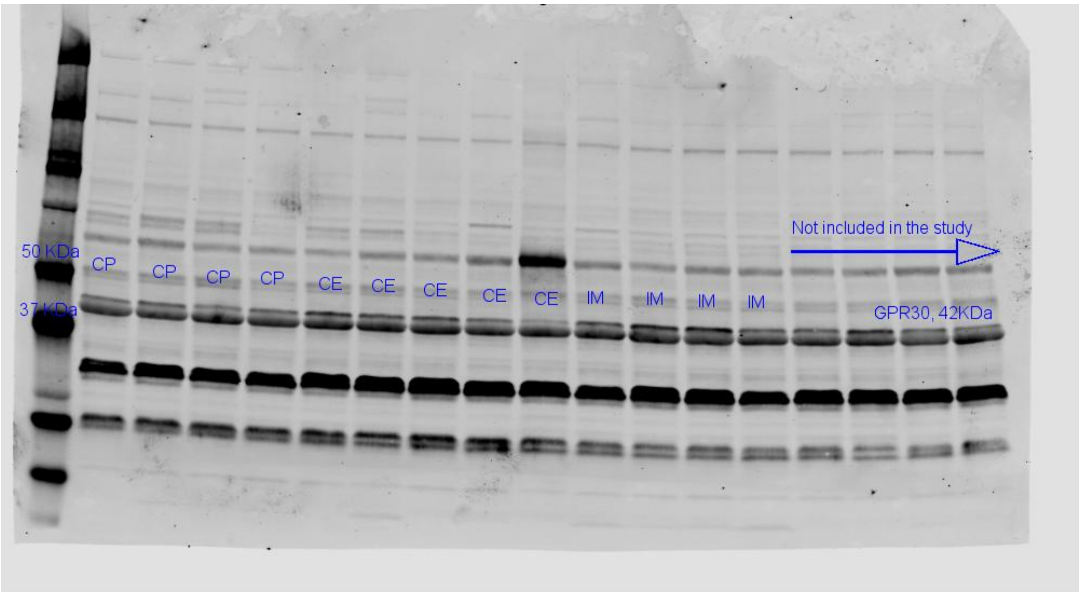

50 KDa
37 KDa
CP
CP
CP
CP
CE
CE
CE
CE
CE
IM
IM
IM
IM
Not included in the study
GPR30, 42KDa

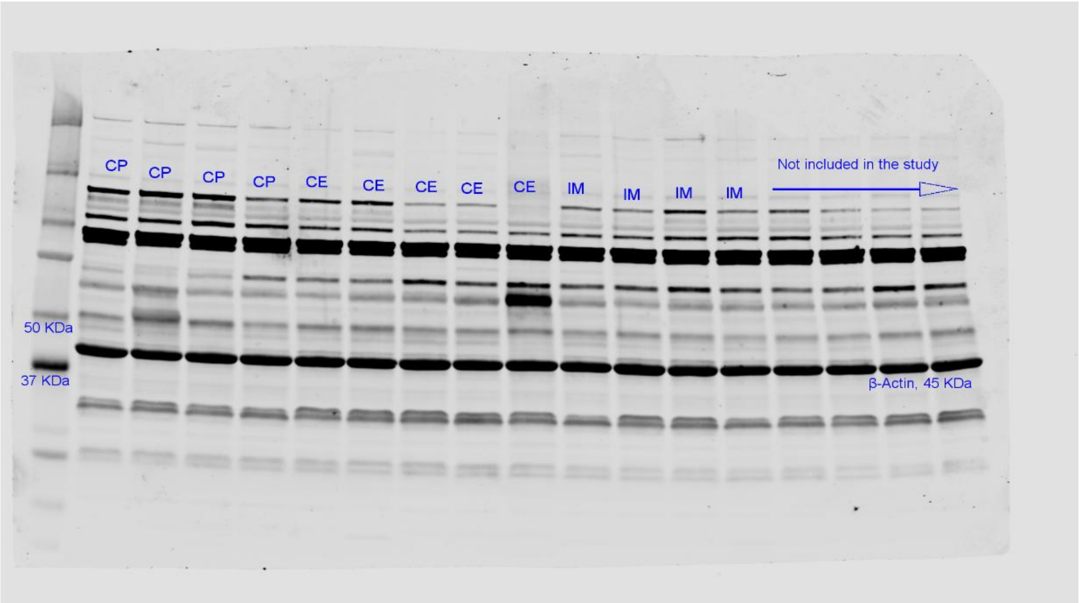

CP
CP
CP
CP
CE
CE
CE
CE
CE
IM
IM
IM
IM
Not included in the study
50 KDa
37 KDa
β-Actin, 45 KDa

D.

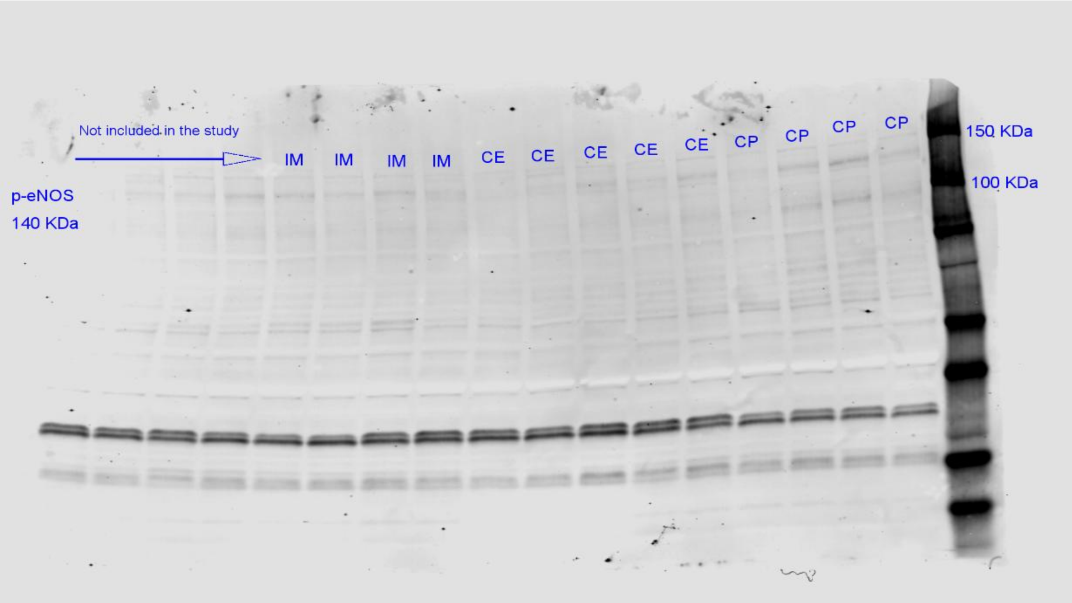
Not included in the study
IM IM IM IM CE CE CE CE CE CP CP CP CP
p-eNOS
140 KDa
150 KDa
100 KDa

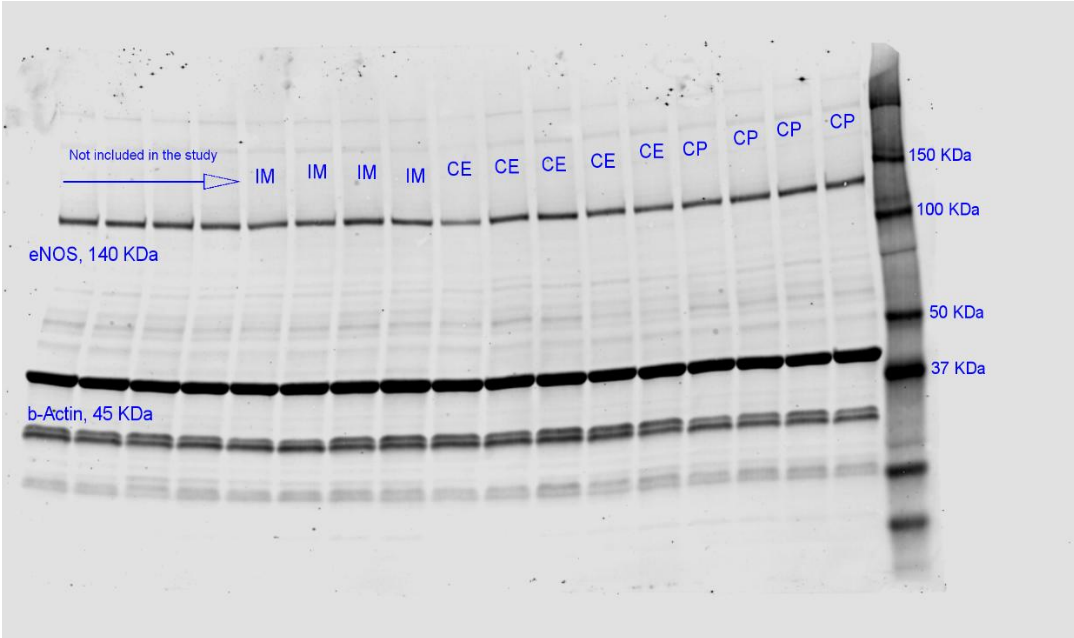
Not included in the study
IM IM IM IM CE CE CE CE CE CP CP CP CP
eNOS, 140 KDa
b-Actin, 45 KDa
150 KDa
100 KDa
50 KDa
37 KDa

E.

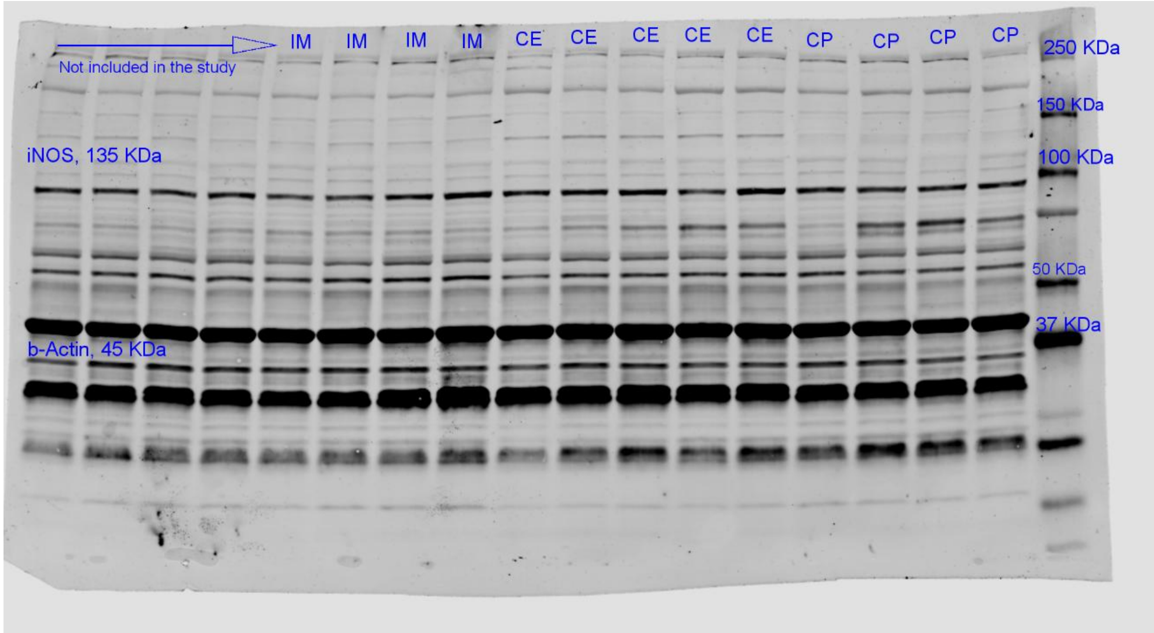

Figure S4. Western blot images of (A) ERα; (B) ERβ; (C) GPR30; (D) p-eNOS and eNOS; (E) iNOS expression in aorta of intact male (IM, n = 4), castrated placebo (CP, n = 4) and CAS E_2-treated (CE, n = 5) rats. Protein levels were quantified by densitometric analysis and normalized to corresponding housekeeping protein. Each lane represents a sample from a different rat. Bands of target and housekeeping proteins were shown from the same membranes. ERα, estrogen receptor α; ERβ, estrogen receptor β; GPR 30, G protein-coupled estrogen receptor 30, p-eNOS, phosphorylated eNOS; iNOS, inducible nitric oxide synthase. Parts of these images were used in **Figures 9A-C and 10A-B** in the Manuscript.
